# The fork protection complex generates DNA topological stress–induced DNA damage while ensuring full and faithful genome duplication

**DOI:** 10.1073/pnas.2413631121

**Published:** 2024-11-26

**Authors:** Andrea Keszthelyi, Sahar Mansoubi, Alex Whale, Jonathan Houseley, Jonathan Baxter

**Affiliations:** ^a^Genome Damage and Stability Centre, School of Life Sciences, University of Sussex, Falmer, Brighton, East Sussex BN1 9RQ, United Kingdom; ^b^Biology Department, North Tehran Branch, Islamic Azad University, Tehran 1477893855, Iran; ^c^Epigenetics Programme The Babraham Institute, Babraham Research Campus, Cambridge CB22 3AT, United Kingdom

**Keywords:** DNA topology, replication stress, DNA damage, topoisomerase

## Abstract

The fork protection complex (FPC), composed of Mrc1, Tof1, and Csm3, supports rapid and stable DNA replication. Here, we show that FPC activity also introduces DNA damage by increasing DNA topological stress during replication. Mrc1 action increases DNA topological stress during plasmid replication, while Mrc1 or Tof1 activity causes replication stress and DNA damage within topologically constrained regions. We show that the recruitment of Top1 to the fork by Tof1 suppresses the DNA damage generated in these loci. While FPC activity introduces some DNA damage due to increased topological stress, the FPC is also necessary to prevent DNA damage in long replicons across the genome, indicating that the FPC is required for complete and faithful genome duplication. We conclude that FPC regulation must balance ensuring full genome duplication through rapid replication with minimizing the consequential DNA topological stress–induced DNA damage caused by rapid replication through constrained regions.

## Significance

The problems inherent in duplicating the eukaryotic genome cause DNA damage during DNA replication. These problems, which stress ongoing replication, are amplified when cells become cancerous. The fork protection complex (FPC) helps to protect cells from different forms of replication stress by stabilizing the replication fork, promoting rapid DNA replication and recruiting Top1 to the replisome to relax the DNA topological stress generated by DNA unwinding. In this study, we show that while FPC rapid replication ensures faithful replication of the genome, it also increases DNA topological stress leading to replication problems in architecturally constrained regions, necessitating Top1 recruitment to reduce DNA damage. This highlights how maintaining genome stability requires balancing rapid replication with consequent DNA topological stress-induced replication stress.

Replication stress is induced by numerous endogenous and exogenous contexts which slow or stall ongoing DNA replication (1). In addition to being a common response to chemically induced DNA damage, replication stress is a recognized hallmark of preoncogenic cells (2). Replication stress varies across the genome according to local chromatin context. Eukaryotic genomes contain numerous “fragile sites” which exhibit elevated markers of replication disruption and DNA damage. Chromosome fragility is linked to different types of replication challenges in these loci. Fork stalling is elevated at stable DNA binding protein complexes or alternate DNA base pairing structures (1, 3–6), consistent with these structures impeding ongoing replication. Chromosome breakage is also increased in long replicons, suggesting that the extended time required to duplicate these regions is linked to heightened challenges to replication completion (7).

A potentially potent cause of replication stress in fragile regions is DNA topological stress (8). DNA topological stress is generated wherever DNA is unwound. The local separation of DNA strands during unwinding causes compensatory overwinding stress in the flanking DNA. Overwound duplex DNA directly causes replication stress by disrupting strand separation by the replicative helicase. This leads to fork stalling and deleterious processing by nucleases (9). To prevent disruptive accumulation of DNA topological stress all cells express topoisomerases. These enzymes transiently break DNA to relieve stress before religation (10). In eukaryotes, the type IB enzyme Top1 and the type II enzyme Top2 act ahead of the replication fork to relax DNA topological stress and prevent replication stalling (8, 9, 11). An alternate pathway to relax DNA topological stress is through the rotation of the whole replication fork relative to the DNA fiber (8). Fork rotation converts overwinding ahead of the fork into sister chromatid intertwines behind the fork. These must then be removed by the double-strand passage action of Top2 before the sister chromatids can be segregated (10).

DNA topological stress has been frequently linked to replication stress and genome instability. DNA boundary sites (e.g., CTCF (CCCTC-binding factor) sites) have high levels of Top2 activity and are associated with frequent DNA breakage and cancer-causing chromosomal translocations (12–15). Focused Top2 activity at these loci indicates that they are barriers to DNA topological stress diffusion and therefore accumulate elevated levels of DNA topological stress (8, 12). Furthermore, the accumulation of cohesin at these sites has been linked to DNA topological stress–induced replication stress in both yeast and human cells (16, 17). Large and stable protein–DNA complexes and nuclear pore attachment sites have also been proposed to induce replication stress by preventing diffusion of DNA topological stress (8, 18, 19). DNA topological stress has also been associated with genome instability in cells with abnormally high levels of replication origin usage via oncogene expression (8, 12, 20, 21). In these cells, the increase in simultaneously elongating replication forks is proposed to overwhelm normal levels of topoisomerase activity leading to impeded fork progression (8, 12, 20, 21).

To prevent frequent fork stalling, sufficient topoisomerase activity must be locally available to rapidly resolve DNA topological stress as it is generated by DNA unwinding (19). In the budding yeast *Saccharomyces cerevisiae* the effective activity of topoisomerases at the replication fork is promoted by a direct interaction between Top1 and the C terminal tail of the evolutionarily conserved replisome factor Tof1 [human (*H.s.*) Timeless] (19, 22–24). Consistent with the notion that topoisomerase recruitment to the replication fork is required to prevent high levels of replication stress, loss of Tof1 or its *H.s.* homolog Timeless increases the constitutive cellular levels of spontaneous DNA damage (19, 25). However, directly connecting DNA damage in Tof1/Timeless depleted cells to changes in topoisomerase activity at the fork is complicated by the fact that Tof1/Timeless is part of the evolutionarily conserved fork protection complex (FPC), which has several other roles in protecting cells from genome instability (26).

The FPC consists of three evolutionarily conserved replication proteins *S.c.* Tof1/*H.s.*Timeless, *S.c.* Csm3/*H.s.*Tipin, and *S.c.* Mrc1/*H.s.*Claspin. Tof1/Timeless and Csm3/Tipin form a heterodimer which interacts with the front face of the CMG helicase and the minor grove of the template DNA (27, 28). This heterodimer interacts with and stabilizes the replisome association of the third FPC factor Mrc1/Claspin (27–29). Together the FPC has multiple reported interactions with both core replication factors, including MCMs 2, 4, 6, 7, Cdc45, AND-1, Rpa1, and Pol Epsilon, and also noncore factors such as Top1, DDX11, Cdc7, PARP1, SDE2, and Spt16 (24, 27, 28, 30–38).

The FPC has multiple functions in promoting rapid and stable DNA replication. It is required for rapid replication fork progression both in vitro and in vivo, stabilizing the replisome under conditions of replication stress and mediating DNA replication checkpoint signaling (26, 39). Of the three FPC factors, loss of Mrc1 leads to the strongest disruption of these processes. Loss of Mrc1 function results in slower replication forks than loss of Tof1 and higher levels of constitutive DNA damage in cells (40–42). However, deletion of either *TOF1 o*r *CSM3* can also disrupt DNA replication independently of Mrc1. Tof1-Csm3 (Swi1-Swi3 in *Schizosaccharomyces pombe*) promotes fork pausing at a variety of protein–DNA complexes independently of Mrc1 function (40, 43–45). Additionally rapid resolution of DNA topological stress at the fork, via Top1 recruitment does not require Mrc1 (19).

Therefore, Tof1/Timeless could be protecting cells from replication stress through FPC complex dependent or independent pathways. Independently of Mrc1 it could prevent the accumulated DNA topological stress stalling the fork (*SI Appendix*, Fig. S1*A*). By regulating fork pausing it could prevent deleteriously rapid passage through impeding structures (*SI Appendix*, Fig. S1*B*). Alternatively, by working with Mrc1, Tof1 could ensure general stability of the replisome as part of the FPC (*SI Appendix*, Fig. S1*C*). Each of these different models of how Tof1 prevents DNA damage makes distinct predictions as to where in the genome replication stress occurs in *tof1Δ* cells and whether DNA damage in the same area is similarly altered in *mrc1Δ* cells. If Tof1 is required to prevent replication stress caused by DNA overwinding, we predict that loss of Tof1 function would increase fork stalling in regions susceptible to DNA topological stress, such as the centromeres and the ribosomal DNA (rDNA) (*SI Appendix*, Fig. S1*A*) (16). If loss of Tof1-dependent fork pausing at DNA-bound structures resulted in replication stress, we would predict that increased replication stress markers would be observed in *tof1Δ* cells where fork pausing occurs. (*SI Appendix*, Fig. S1*B*). If absence of Tof1 causes DNA damage due to loss of a Mrc1-Tof1 linked FPC function, we would expect replication to be defective more generally. We would also predict that loss of Mrc1 would similarly cause damage in these regions (*SI Appendix*, Fig. S1*C*).

Here, we test these models to determine which chromosomal contexts are most closely linked to increased replication related DNA damage in *tof1Δ, mrc1Δ* or cells defective in recruiting Top1 to the fork.

## Results

### A Tof1- and Mrc1-Dependent FPC Function Causes DNA Damage at Centromeres and the rDNA.

In *S.c.* the DNA damage sensing kinases Mec1^ATR^ and Tel1^ATM^ phosphorylate histone H2A at Serine 129 to generate H2AS129P (H2AP—γH2AX in human cells) (46). Mec1^ATR^ is active at sites of replication stress (47) due to local exposure of single stranded DNA (ssDNA) (*SI Appendix*, Fig. S1 *A*–*C*) (48). Constitutive sites of replication stress in yeast have been identified by genome-wide chromatin immunoprecipitation (ChIP) of H2AP (4). Therefore, to determine where in the yeast genome loss of Tof1 caused replication stress, we compared the H2AP ChIP-SEQ profile (49) of *wt* and *tof1Δ* cells. We first confirmed that our H2AP ChIP-SEQ analysis identified the same range of constitutive replication stress-induced sites previously identified by genome-wide H2AP ChIP. In wildtype cells, we confirmed strong enrichment of H2AP at telomeres, the rDNA repeats, the mating type loci HML and HMR, transfer RNA genes (tRNAs), and origins of replication (*SI Appendix*, Fig. S2 *A*–*F*). We observed modest enrichment of H2AP at centromeres and LTR transposons in exponentially growing cells (*SI Appendix*, Fig. S2 *G* and *H*). However, comparison of H2AP in G1 arrested to exponential cycling cell showed that H2AP was specifically enriched in cycling cells. (*SI Appendix*, Fig. S2 *I* and *J*). This is consistent with our previous study that showed that H2AP is specifically enriched during S phase at centromeres (16). We also confirmed that repression of galactose inducible genes increases local H2AP enrichment (4) (*SI Appendix*, Fig. S3 *A* and *B*).

**Fig. 1.**
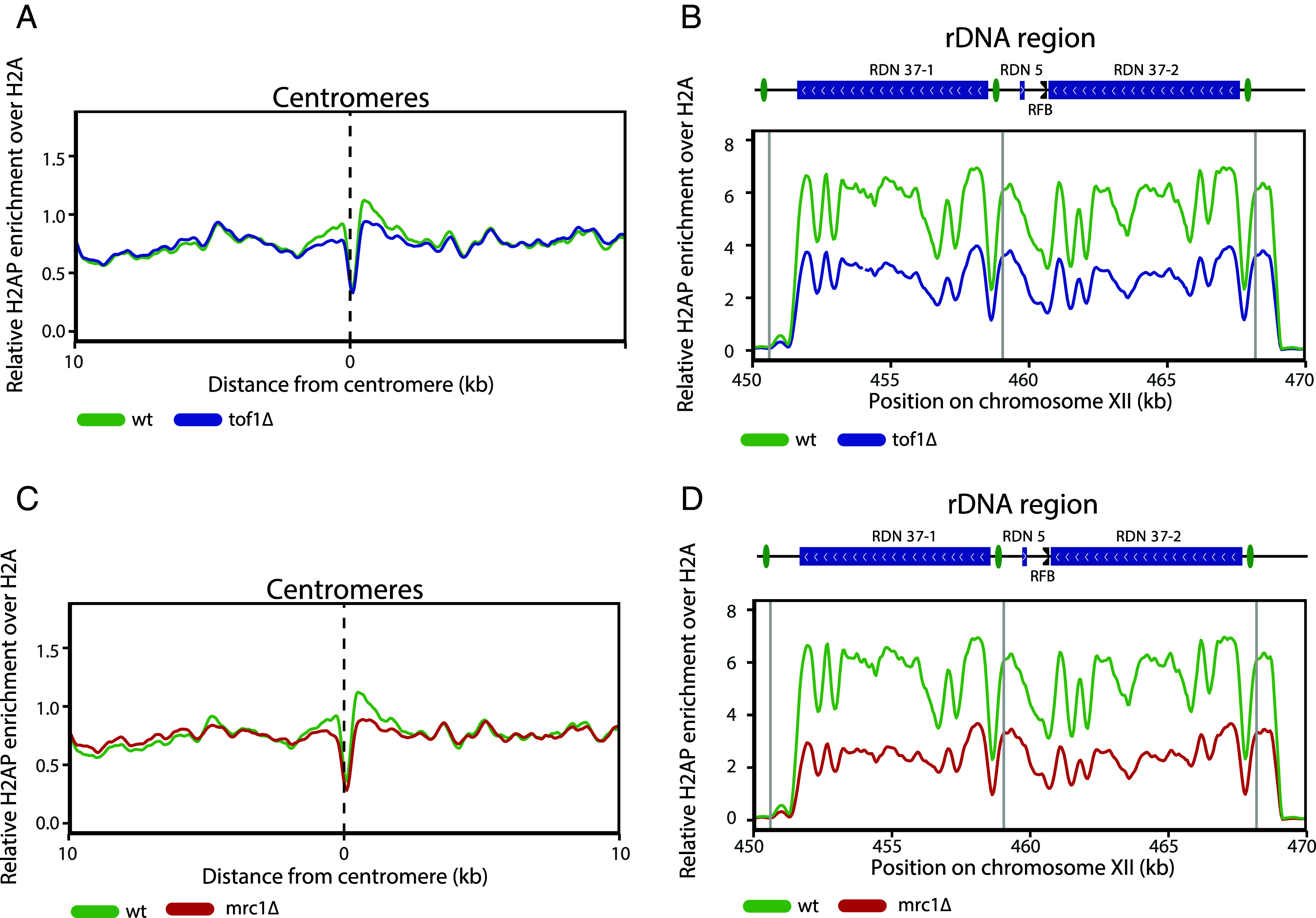
Loss of either Tof1 or Mrc1 decreases DNA damage at centromeres and the rDNA. Relative H2AP enrichment over H2A in *wt* and *tof1∆* cells at (*A*) around centromeres, (*B*) at the rDNA repeats. Relative H2AP enrichment over H2A in *wt* and *mrc1∆* cells at (*C*) around centromeres, (*D*) at the rDNA repeats. Smoothing with moving average over seven bins (50 bp bin size) was applied.

Next, we assayed how the genome-wide distribution of H2AP was altered by loss of Tof1 activity. Since centromeres and the rDNA repeats are sites of both DNA topological stress accumulation, and of Tof1-dependent fork pausing (16, 40, 44, 50), we anticipated that they were likely sites of increased H2AP in *tof1Δ* cells. Surprisingly, we observed reduced H2AP at the centromeres (Fig. 1*A*) and across the rDNA repeats including at the replication fork block (RFB) region in *tof1Δ* cells (Fig. 1*B*). To ensure that loss of H2AP signal across the rDNA was not related to loss of rDNA copy number (*SI Appendix*, Fig. S3*C*), we normalized the counts of H2AP ChIP-SEQ recovered DNA sequences in the rDNA to either unmodified H2A ChIP-SEQ sequence counts (Fig. 1*B*) or to input sequence counts (*SI Appendix*, Fig. S3*D*). Using either approach, we observed a loss of H2AP accumulation in *tof1Δ* cells across the rDNA. To test whether loss of endogenous DNA damage at centromeres and rDNA was related to a Tof1-Mrc1 linked function of the FPC, we also examined changes to H2AP accumulation in *mrc1*Δ cells. Reduced accumulation of H2AP at both centromeres (Fig. 1*C*) and across the rDNA repeats (Fig. 1*D*) was also observed in *mrc1Δ* cells. This indicates that elevated H2AP accumulation at centromeres and the rDNA in *wt* cells is connected to the Mrc1-Tof1 linked functions of the FPC.

### Top1 Recruitment to the Replication Fork Reduces DNA Damage at Centromeres and the rDNA.

**Fig. 2.**
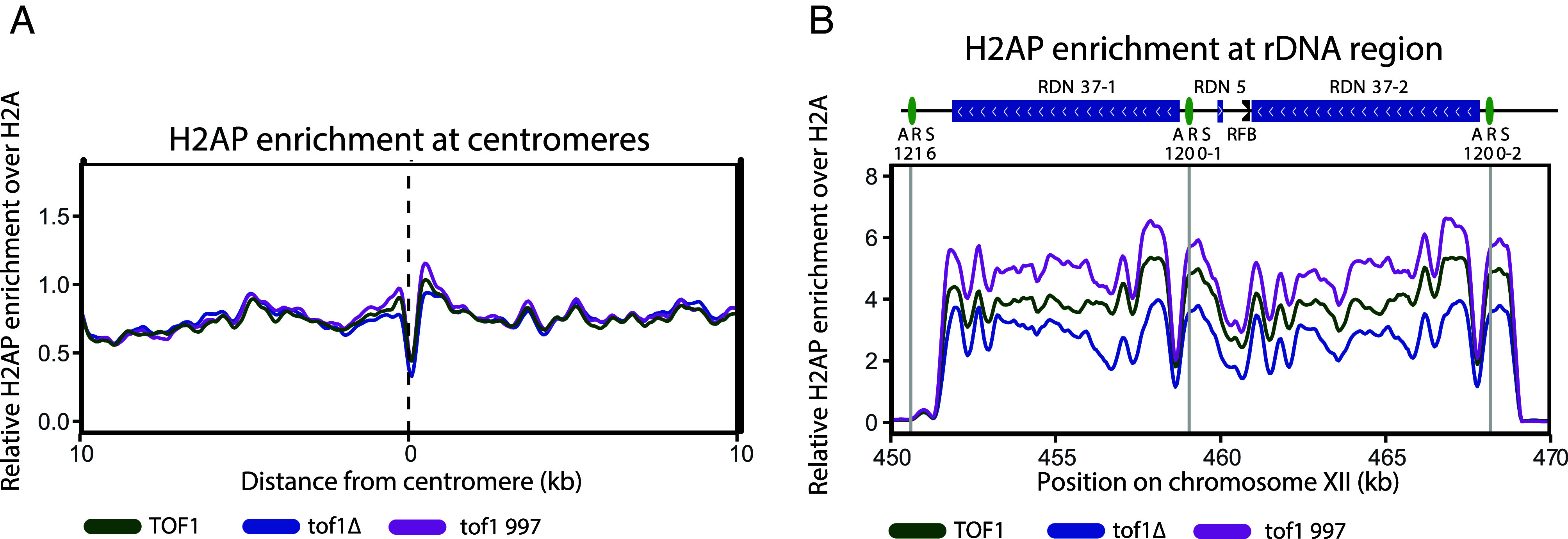
Tof1 recruits Top1 to suppress DNA damage accumulation at the centromeres and rDNA in rapidly replicating FPC+ cells. (*A*) Relative H2AP enrichment in *TOF1wt, tof1∆,* and *tof1 997* cells around centromeres and (*B*) rDNA. The strains were generated by complementing *tof1∆* cells with either *wt TOF1* or *tof1 997*. Smoothing with moving average over 20 bins (50 bp bin size) was applied for (*A*), and seven bins (50 bp bin size) for (*B*).

Since we have previously shown that loss of Top2 results in increased replication dependent-H2AP at centromeres and the rDNA repeats (16), it was surprising that the loss of Tof1, and its activity in resolving DNA topological stress, led to a reduction in DNA damage at these regions. However, the observed loss of H2AP in both *tof1Δ* and *mrc1Δ* suggested this change was due to loss of a core FPC function and not specifically related to loss of Top1 recruitment to the fork. To directly test the role of Top1 recruitment by Tof1 we assayed the H2AP/H2A ChIP-SEQ of *tof1 997* expressing cells (49). The *tof1 997* mutant is proficient in fork pausing and replication checkpoint activation but does not interact with Top1 (22). In *tof1 997* cells, we observed increased H2AP around centromeres (Fig. 2*A*) and across the rDNA repeats (Fig. 2*B*). This indicates that Top1 recruitment to the fork is required to reduce H2AP accumulation at these loci, if core FPC functions, required for rapid and stable DNA replication, are maintained.

Together these data support an updated model of Tof1 action on DNA topological stress. In this revised model recruitment of Top1 to the fork by Tof1 is important to prevent replication stress at centromeres and the rDNA when Tof1-Mrc1 supported rapid replication is occurring (*SI Appendix*, Fig. S4*A*). When Tof1 and Mrc1 are functional rapid and stable replication provided by the FPC increases DNA topological stress across the centromeric and rDNA regions to a point where the direct recruitment of Top1 to the fork is required to prevent fork stalling and DNA damage (*SI Appendix*, Fig. S4 *A*, *Center* to *Right*). In contrast, in the absence of FPC activity (in *tof1Δ* and *mrc1Δ* cells), the resulting slow replication does not rapidly accumulate DNA topological stress (*SI Appendix*, Fig. S4 *A*, *Center* to *Left*). Therefore, in this model recruitment of Top1 to the fork is not required to prevent fork stalling when the FPC is not stimulating rapid and stable DNA replication.

### Mrc1 Activity Increases DNA Topological Stress Ahead of the Replication Fork on Plasmids.

**Fig. 3.**
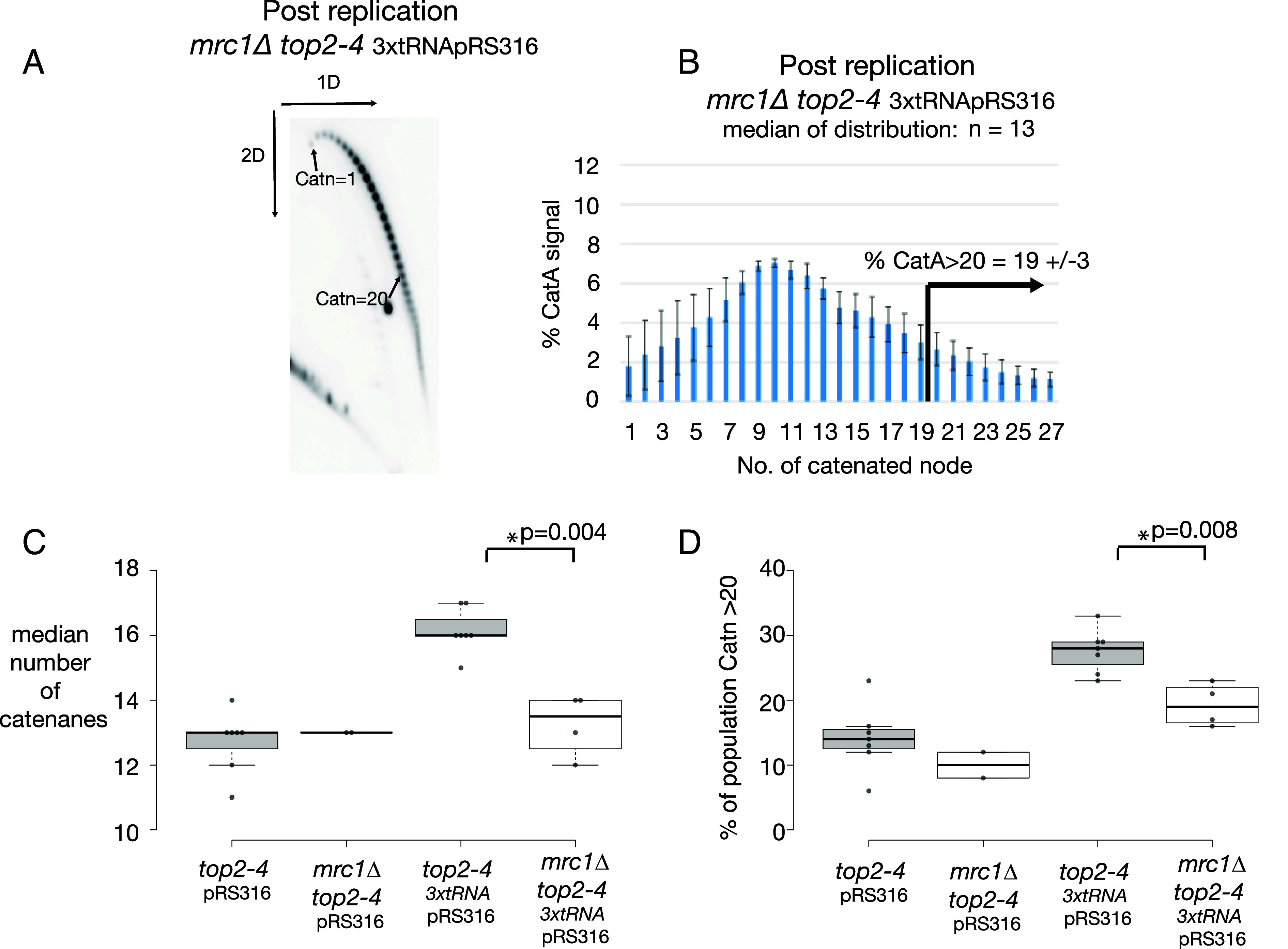
Loss of Mrc1 reduces the level of DNA topological stress ahead of the replication fork during replication of the *3xtRNApRS316* plasmid. The frequency of fork rotation in S phase in *mrc1∆ top2-4 3xtRNApRS316* (*Top*) was assessed by analyzing DNA catenation on the plasmid following one round of DNA replication in the absence of Top2 activity. (*A*) First DNA catenanes were separated on a first dimension of agarose gel electrophoresis (1D), before full resolution using a 2nd dimension of agarose gel electrophoresis (2D). A representative autoradiogram is shown. (*B*) The relative intensity of catenanes generated post replication was quantified and the population median of the catenanes and the % of plasmids with >20 catenanes (Catn>20) calculated for each of the conditions. The histogram shows the relative average distribution of the intensity of catenanes generated post replication from four individual experiments. Error bars represent the average deviation of the repeats. The ± of the % CatA>20 is the largest deviation from the mean of the four experiments. (*C*) The medians from each of the four individual experiment of *mrc1∆ top2-4 3xtRNApRS316* averaged in (*B)* are plotted individually (black dots) and overlaid with a box plot format and compared to the level of catenanes from the same assessment of *top2-4 pRS316* (six repeats) *mrc1∆ top2-4pRS316* (two repeats) and *top2-4 3xtRNA pRS316* (six repeats). The median of each experiment (horizontal black line) is plotted on the box plot with the boxes representing the middle two quartiles of the distributions of the dataset. *P*-values are derived from paired *t* tests, a star indicates a significant difference between two conditions (*P* < 0.05). (*D*) The % of plasmids with >20 catenanes (Catn>20) from each individual experiment shown in panel (*C*), are replotted in box plot format as in *C*.

Although the revised model is fully consistent with our data, the data do not rule out other potential explanations. For example, because the Tof1 C terminal region that recruits Top1 extensively overlaps with the region that interacts with Spt16 (51), it is possible that disruption of chromatin remodeling ahead of the fork could generate the observed pattern of DNA damage. Since it is not currently plausible to alter Tof1 to singularly disrupt Top1 recruitment, we instead assayed alternative predictions of this hypothesis. The model predicts that Mrc1-promoted rapid replication will generate high levels of DNA topological stress in chromosomal contexts where stress diffusion is limited. Previously, we have utilized a plasmid-based assay to measure the extent of DNA topological stress imposed on replication in different genetic contexts (16, 19). In this assay, genetic contexts that increase replication-dependent topological stress increase the frequency of fork rotation during elongation, resulting in increased catenation of the plasmid following replication. In cells where decatenation activity has been ablated, the number of DNA catenanes formed during replication of the plasmids in vivo can be directly assayed by two-dimensional agarose gel electrophoresis and Southern blotting (19). Increased frequency of highly catenated plasmids in the population indicates increased DNA topological stress accumulation during their replication. Decreased number of DNA catenanes indicate reduced DNA topological stress accumulation ahead of the fork during plasmid duplication (19). If the model (*SI Appendix*, Fig. S4*A*) is correct, we would predict that the loss of FPC function caused by *mrc1Δ* should reduce DNA topological stress ahead of the fork, leading to a lower frequency of DNA catenanes being generated on the duplicated plasmid. We have previously analyzed the plasmid catenation in *mrc1Δ* cells on the centromeric plasmid pRS316 and observed a nonsignificant reduction in plasmid catenation (19) (Fig. 3). However, in our previous study, we also concluded that most fork rotation on this plasmid occurs during the termination of DNA replication, with only a relatively small contribution of catenation from elongation occurring (during replication through the centromere) (19). To better examine the effect of loss of Mrc1 on fork rotation during elongation we examined the extent of fork rotation on the plasmid 3xtRNApRS316 (Fig. 3 *A* and *B*). This plasmid has previously shown to undergo more fork rotation during elongation due to the presence of three tRNA genes (3xtRNA) generating higher levels of DNA topological stress accumulation during elongation (19). Using this plasmid, MRC1+ cells generate catenated plasmid populations with a median of 16 catenanes, with 28% of the population with >20 catenanes (19) (Fig. 3 *C* and *D*). However, in *mrc1Δ* cells we observed a significant reduction in fork rotation during DNA replication, with catenated plasmid populations with a median of 13 catenanes, and 19% of the population with >20 catenanes (Fig. 3 *C* and *D*). Therefore, loss of Mrc1 activity reduces the extent of fork rotation produced by DNA topological stress on this plasmid. In addition to showing that Mrc1 contributes to replication-induced DNA topological stress on the CEN and tRNA containing 3xtRNApRS316 plasmid, these data also suggest that centromeres flanked by tRNA on linear chromosomes are more likely to be difficult to replicate than centromeres without bordering tRNA. Meta analysis of H2AP at centromeres with proximal tRNAs showed that centromeres with neighboring tRNA accumulate higher H2AP (*SI Appendix*, Fig. S4*B*), than centromeres not associated with tRNA (*SI Appendix*, Fig. S4*C*), consistent with this interpretation.

**Fig. 4.**
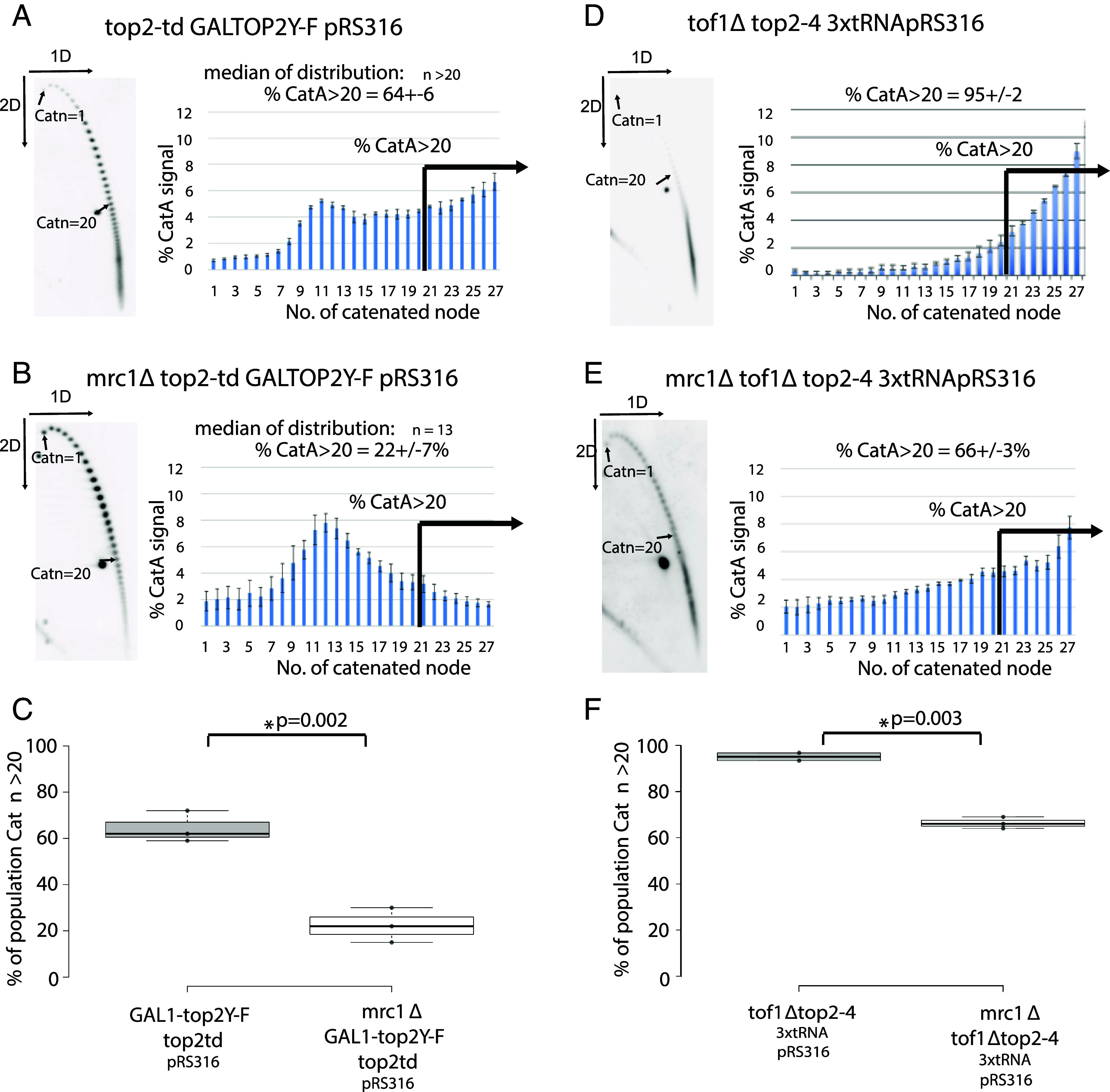
Loss of Mrc1 reduces the level of DNA topological stress ahead of the replication fork during replication of plasmids. The frequency of fork rotation in S phase in (*A) GAL1-top2Y-F top2td pRS316* and (*B*) *mrc1∆ GAL1-top2Y-F top2td pRS316* were assessed as described in Fig. 3*B*. Histograms showing the relative distribution of the intensity of catenanes generated in each strain type were quantified and calculated from the average of three individual experiments Error bars represent the average deviation of node intensity of the repeats. The ± of the % CatA>20 is the largest deviation from the mean of the individual experiments. (*C*) The % of plasmids with >20 catenanes (Catn>20) from each individual experiment (black dots) used in *A* and *B* are directly compared in box plot format as described in Fig. 3*C*. Fork rotation in (*D*) tof1Δ top2-4 3xtRNApRS316 and (*E*) mrc1Δ tof1Δ top2-4 3xtRNApRS316 cells were assessed as described in Fig. 3*B*. Two individual experiments were assessed for D, three individual experiments for E. (*F*) The % of plasmids with >20 catenanes (Catn>20) from each individual experiment used in *D* and *E* are directly compared in box plot format as described in *C*.

To ascertain whether reduced fork rotation in *mrc1Δ* cells was specific to the CEN tRNA plasmid context of 3xtRNApRS316, or was observed more generally during DNA replication, we examined how *mrc1Δ* altered fork rotation in other situations engineered to generate high levels of DNA topological stress ahead of the fork. Expression of enzymatically inactive Top2 (Top2Y-F) in cells depleted of wildtype Top2 delays termination of DNA replication and causes high levels of fork rotation on plasmids during elongation (52). This phenotype is likely due to competitive inhibition of Top1 by the physically present but inactive Top2Y-F protein on DNA topological stress ahead of the fork (8). We deleted *MRC1* in *GAL1TOP2Y-F top2-td pRS316* cells, and then collected DNA from cells after one round of DNA replication, following depletion of active Top2 and expression of the catalytically inactive Top2Y-F protein (52). Analysis of MRC1+ cells showed that normal FPC activity in these cells produced a catenated plasmid population with 64% of the pRS316 plasmids having greater than 20 catenated crossing (Catn >20) (Fig. 4 *A* and *C*). Deletion of *MRC1* in these cells caused a dramatic reduction in the extent of plasmid catenation resulting in only 22% of the pRS316 population having >20 catenanes following replication (Fig. 4 *B* and *C*). To ensure the expression of Top2Y-F in *mrc1Δ* was maintained to the same level as *MRC1*+ cells, we assessed Top2Y-F expression in both *MRC1+* and *mrc1Δ* cells. The heavily modified Top2Y-F protein (52) was expressed to similar level in both backgrounds (*SI Appendix*, Fig. S5). We conclude that loss of Mrc1 activity strongly reduces the levels of DNA topological stress at the fork caused by expression of the Top2Y-F protein.

Finally, we examined the effects of the different losses of fork speed in *mrc1Δ and tof1Δ* cells on the extensive plasmid catenation caused by loss of Top1 recruitment to the fork. Plasmids become hypercatenated in *tof1Δ* cells depleted of Top2 activity (19) due to loss of recruitment of Top1 to the fork (22, 23). This shows that, at least on circular plasmids, even relatively slow replication forks require direct recruitment of Top1 to the fork to prevent extensive precatenation (40, 41). However, our model predicted that expression of a *tof1* mutant predicted to maintain rapid replication but deficient for Top1 recruitment should restore rapid replication of these plasmids and therefore cause higher levels of hypercatenation than the null *tof1Δ* mutation. Visual comparisons of the extent of catenation in *tof1 997 top2-4* (where replication speed is predicted to be faster) versus *tof1Δ top2-4* cells (where replication is predicted to be slower) from our previously published studies were consistent with this model (19, 22). However, the extreme levels of hypercatenation in *tof1 997 top2-4* precluded accurate quantification of this difference. Therefore, to perform a comparable, quantifiable experiment, we compared the extent of fork rotation and DNA catenation on 3xtRNApRS316 plasmids in *mrc1Δ tof1Δ top2-4* cells relative to *tof1Δ top2-4.* The deletion of both *MRC1* and *TOF1* produces slower DNA replication than deletion of *TOF1* alone (41, 53). Therefore, our model predicts that *mrc1Δ tof1Δ* cells should generate less DNA topological stress ahead of the fork than *tof1Δ* cells. This predicts that hypercatenation in *mrc1Δ tof1Δ top2-4* will be lower than on plasmids from *tof1Δ top2-4* cells. We found that *mrc1Δ tof1Δ top2-4* cells plasmids were 66% hypercatenated (>20 catenanes) compared to 95% of cells being hypercatenated following replication in *tof1Δ top2-4* (Fig. 4 *D*–*F*). This demonstrated that the DNA topological stress ahead of the fork is lower in *mrc1Δ tof1Δ* cells than in *tof1Δ* cells, as predicted by the model.

We conclude from these distinct approaches to assessing DNA topological stress during DNA replication, that the rapid replication fork produced by Mrc1 causes high levels of DNA topological stress ahead of the replication fork.

### Deletion of Tof1 Does Not Increase DNA Damage at Fork Pausing Sites Confirmed by TrAEL-SEQ.

**Fig. 5.**
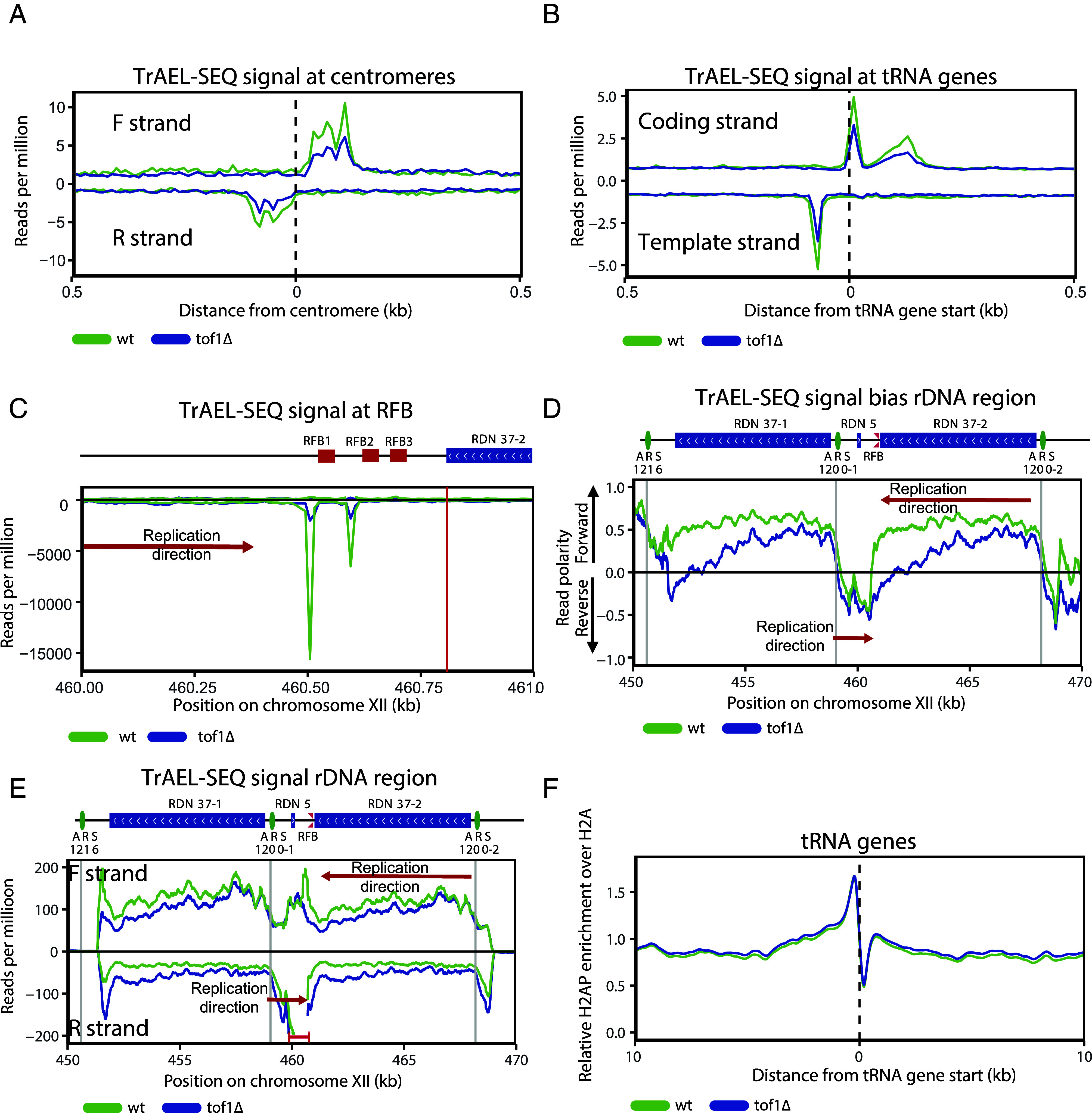
Tof1-dependent fork pausing and DNA damage do not correlate genome-wide. Directional TrAEL-SEQ signal at (*A*) tRNA genes, (*B*) centromeres, (*C*) rDNA replication fork barrier normalized for the different rDNA copy number of *wt* and *tof1∆* cells, (*D*) Ratio of TrAEL-SEQ signal on forward and reverse strand (read polarity) and (*E*) Directional TrAEL-SEQ signal over the entire rDNA region, normalized for the difference in rDNA copy number between *wt* and *tof1∆* cells. Gray vertical lines indicate positions ofautonomously replicating sequence (ARS) sequences in the region. For (*A*–*C)*, no moving average was applied (bin size 10 bp). For (*E*) moving average over 20 bins (10 bp bin size) was applied. In *E* within the region bracketed in red, reads exceed the y axis. This region is shown to a full scale in panel (*C*). Red arrows labeled replication direction mark expected direction of DNA replication over selected regions in wildtype cells. (*F*) Relative H2AP enrichment over H2A in *wt* and *tof1∆* cells centered on start of tRNA genes. Smoothing with moving average over seven bins (50 bp bin size) was applied.

Our finding that Mrc1/FPC action increased DNA topological stress accounts for the reduction in DNA damage observed at centromeres and the rDNA repeats in *mrc1∆* and *tof1∆* cells. To ascertain whether there are other loci in the genome where Tof1’s role in enforcing replication fork pausing is preventing rather than exasperating DNA damage, we first sought to globally identify Tof1-dependent replication pause sites. To achieve this, we used the TrAEL-SEQ (Transferase-Activated End Ligation sequencing) assay to assess genome-wide replication dynamics of *wt* and *tof1∆* cells (54). The TrAEL-SEQ technique detects 3′ nascent DNA ends on the leading strand of elongating replication forks and is particularly sensitive to 3′ ends exposed at reversed forks. Previous analysis of wild type cells has shown that TrAEL-SEQ signal is strongly elevated at replication pausing sites across the *S.c*. genome (54), including the RFB, centromeres, and tRNA genes. TrAEL-SEQ is strand sensitive and therefore also provides information on directional bias at pause sites. In *tof1Δ* cells, we observed reduction of TrAEL-SEQ signal (49) at centromeres and tRNAs genome-wide (Fig. 5 *A* and *B*), consistent with previously reported loss of fork pausing at individual centromere and tRNAs in *tof1Δ* cells (50). However, TrAEL-SEQ also demonstrated further detail on the nature of Tof1-dependent fork pausing at tRNA. In wildtype cells, TrAEL-SEQ signal is detected both upstream and downstream of the tRNA start site (Fig. 5*B*) (54). The accumulation of both these signals was reduced in *tof1Δ* cells (Fig. 5*B*), indicating that Tof1 activity is involved in both pausing contexts, potentially at collisions with promoter proximal and terminating RNA polymerase III complexes.

We also observed a strong, *tof1Δ*-dependent reduction in the unidirectional TrAEL-SEQ signal at the RFB1 and RFB2 sequences of the RFB locus of the rDNA (Fig. 5*C*), consistent with Tof1 activity being required at the RFB to enforce the fork block (40, 44). The RFB normally prevents replication forks replicating the 35S gene in the opposite direction to RNA polymerase I transcription (55). This is reflected by the strong bias of TrAEL-SEQ signal in the forward direction (representing replication forks moving right to left) in wildtype cells across the rDNA outside of the autonomously replicating sequence - Replication fork barrier (ARS-RFB) region (Fig. 5*D*). In *tof1Δ* cells we observe a decrease in this bias, consistent with a population of forks now being able to pass through the RFB (Fig. 5*D*). Examination of the quantity of strand-specific TrAEL-SEQ signals over the rDNA repeats (Fig. 5*E*) (normalized for the difference in rDNA repeat number in *wt* and *tof1Δ* cells—*SI Appendix*, Fig. S3*C*), showed reduced forward (F) strand and increased reverse (R) strand TrAEL-SEQ signal in *tof1Δ.* Since the gain in R strand signal appears to mirror loss in F strand signal, we postulate that the changes in this region are due to the rDNA repeats now being replicated in both directions rather than in a unidirectional manner. However, we cannot rule out that increased TrAEL-SEQ signal in the R direction could be due in part to head on collisions between the *tof1Δ* forks and transcribing RNA polymerase.

From TrAEL-SEQ, we can conclude that loss of Tof1 results in loss of pausing at centromeres, the rDNA RFB, and tRNA genome-wide. However, loss of pausing at these structures did not lead to an increase in replication stress or DNA damage markers at these loci. Our H2AP/H2A ChIP-SEQ analysis showed that loss of Tof1 caused a reduction of H2AP at centromeres and across the rDNA (Fig. 1 *A* and *B*), while at tRNAs we did not detect any changes (Fig. 5*F*).

In summary, we did not observe any support for the model that loss of Tof1-dependent fork pausing contributes to increased replication stress or DNA damage.

### Tof1 and Mrc1 Protect Long Replicons from DNA Damage during S Phase.

**Fig. 6.**
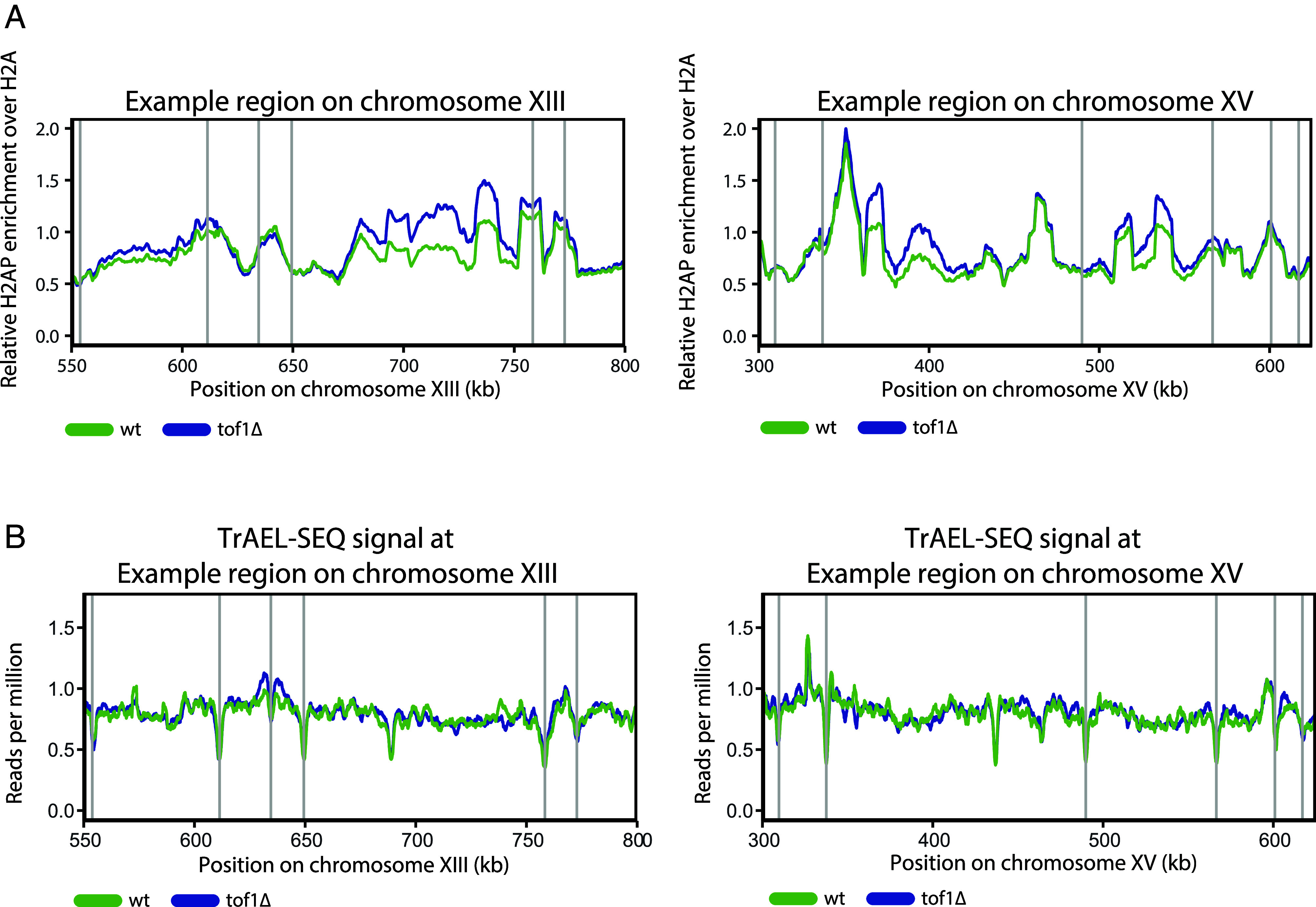
DNA damage in *tof1∆* cells accumulates in regions with relatively few origin sites and is not linked to pausing sites. (*A*) Relative H2AP enrichment over H2A in *wt* and *tof1∆* cells at two example regions on chromosome XIII (*Left*) and XV (*Right*) where H2AP signal was elevated. Smoothing with moving average over seven bins (50 bp bin size) was applied. (*B*) Cumulative (forward and reverse strand) TrAEL-SEQ signal at two example regions on chromosome XIII (*Left*) and XV (*Right*). Gray vertical lines indicate positions of ARS sequences in the region, moving average over 200 bins (10 bp bin size) was applied.

Since we did not observe increased H2AP at candidate genomic sites, we carried out a visual inspection of H2AP across individual yeast chromosomes. We observed regional changes in H2AP on several chromosomes. Two examples are shown in Fig. 6*A*. Examination of TrAEL-SEQ signal across these H2AP-enriched regions did not support any connection between local fork pausing and H2AP accumulation (Fig. 6 *A* and *B*). Rather, it suggested a link between a relative lack of ARS sequences and H2AP signal (Fig. 6*A*).

**Fig. 7.**
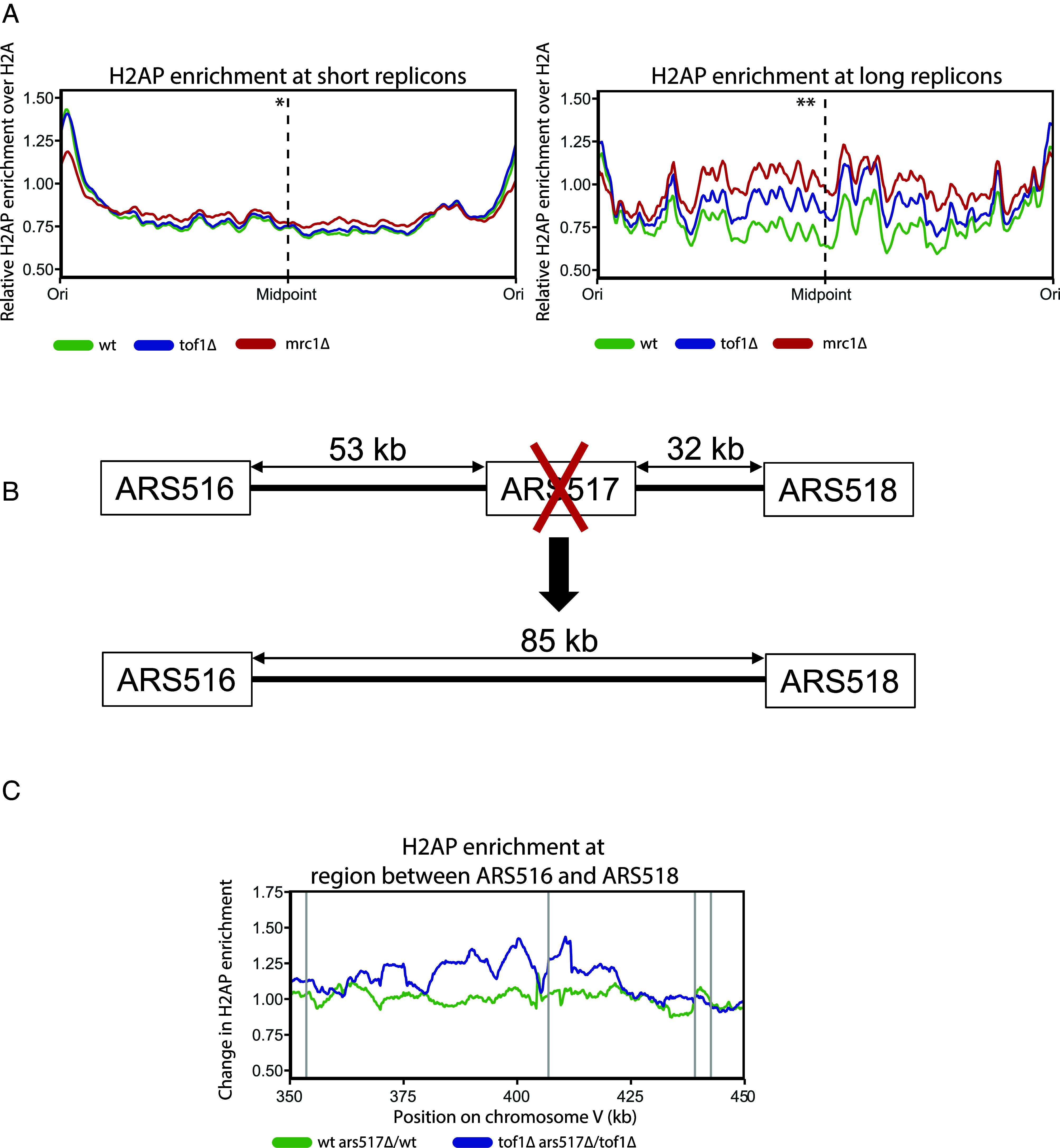
Tof1 protects long replicons from DNA replication stress. (*A*) Relative H2AP enrichment in *wt, tof1∆,* and *mrc1∆* cells over short replicons (20 kb to 50 kb) (*Left*) and long replicons (>60 kb) (*Right*). *P-value based on *t* test on the average of the middle 10 bins for *tof1∆* against *wt* in short replicons: = 0.8688. **P-value of *tof1∆* against *wt* in long replicons: = 0.004. (*B*) Schematic representation of the conversion of two relatively short replicons into one long replicon. (*C*) Ratio between relative H2AP enrichment in the presence or absence of ARS517 origin in *wt* and *tof1∆* cells. Gray vertical lines indicate positions of ARS sequences in the region. Smoothing with moving average over 20 bins (50 bp bin size) for (*A*) and 100 bins (50 bp bin size) for (*C*) was applied.

A regional absence of ARS sequences is associated with longer replicons. To test the hypothesis that replication stress in *tof1Δ* cells was preferentially occurring in longer replicons, we took all ARS sequences assessed as likely to fire in most cell cycles (efficiency >40—based on ref. 56) and used these sites to subdivide the genome into regions either likely replicated as part of a short replicon (20 kb to 50 kb) or a long replicon (>60 kb) (57). We then assessed the average change in H2AP across different replicon sizes in *wt* and *tof1Δ* cells. Loss of Tof1 (*tof1Δ*) causes a marked increase in H2AP in long replicons while showing little effect in short replicons (Fig. 7*A*). Using our H2AP/H2A ChIP-SEQ data from *mrc1Δ* cells (49) we also observed strong accumulation of H2AP in the same long replicons affected in *tof1Δ* cells, with relatively minimal accumulation of H2AP in short replicons (Fig. 7*A*). H2AP signal distribution in the affected regions of *mrc1Δ* cells was very similar to the profile of *tof1Δ* cells, but with a notably higher intensity toward the middle of the replicons (Fig. 7*A*). This indicates that loss of Mrc1 disrupts DNA replication in the same long replicons as Tof1, but in a more penetrative fashion.

To directly test the link between replicon length and DNA damage in *tof1Δ* cells, we converted two relatively short replicons, that did not accumulate DNA damage in *tof1Δ* cells, into one long replicon (Fig. 7*B*). If loss of Tof1 was specifically leading to DNA damage in long replicons, then the generation of a long replicon should specifically lead to accumulation of H2AP in *tof1Δ* cells but not in wildtype cells. We deleted the active early origin ARS517, to convert the two replicons between ARS516 to ARS517 and ARS517 to ARS518 into one large replicon extending from ARS516 to ARS518 (Fig. 7*B*). Comparing cells with and without ARS517 we observed that extension of replicon size had little effect on H2AP enrichment in wildtype cells (Fig. 7*C*). In contrast, in *tof1Δ* cells the generation of an expanded replicon caused a marked accumulation of H2AP (Fig. 7*C*). This shows that the accumulation of replication stress markers in *tof1Δ* cells in longer replicons is not primarily dependent on underlying sequence. Rather, it demonstrates that replication stress in *tof1Δ* cells is a result of increased distance between origins of replication.

### Persistent DNA Damage and ssDNA Accumulate in Long Replicons in the Absence of Mrc1.

To further characterize the DNA damage generated by FPC inactivation and the apparent difference in the intensity of DNA damage in long replicons in *mrc1Δ* cells relative to *tof1Δ*, we examined cell cycle variation in H2AP accumulation. Although H2AP accumulated in *tof1Δ* exponentially growing cells, this was not observed in either G1 synchronized (treated with alpha factor) or mitotically arrested cells (arrested with nocodazole) (*SI Appendix*, Fig. S6*A*). This argues that *tof1Δ* cells accumulate H2AP primarily in S phase and that the associated DNA lesions are not maintained into mitosis. In contrast, H2AP was elevated in *mrc1Δ* cells in both exponential and mitotic (postreplicative) cells, while displaying similar levels to *wt* cells in G1 (*SI Appendix*, Fig. S6*A*). This indicates that DNA lesions generated during S phase in *mrc1Δ* cells either accumulate to a level where DNA repair kinetics are insufficient to remove lesions before entry into mitosis, or the loss of the checkpoint signaling functions of Mrc1 results in delayed repair. Either scenario is consistent with the *RAD9-*dependent DNA damage pathway being essential for survival in *mrc1Δ* cells (42).

Prior studies have shown that both Tof1 and Mrc1 are required to prevent uncoupling of helicase and polymerase activities when cells are subjected to exogenous replication stress-inducing agents (58). Uncoupled regions are marked by relatively long stretches of ssDNA and increased chromatin binding of the ssDNA binding protein complex RPA (which is composed of Rfa1, Rfa2, and Rfa3 in *S.c.*). To determine whether increased exposure of ssDNA is a feature of the DNA damage that accumulates in long replicons in *mrc1Δ* and *tof1Δ* cells, we performed Rfa1-ChIP in exponentially growing *wt*, *mrc1Δ* and *tof1Δ* cells (49). In *tof1Δ* cells Rfa1 ChIP-SEQ showed no significant accumulation of Rfa1 across long replicons (*SI Appendix*, Fig. S6*B*). In contrast, in *mrc1Δ* cells we observed strong accumulation of Rfa1 ChIP-SEQ signal, primarily in the central regions of long replicons (*SI Appendix*, Fig. S6*B*). Increased Rfa1 ChIP-SEQ signal was not observed in short replicons of *mrc1Δ* cells (*SI Appendix*, Fig. S6*B*). Therefore, markers of fork uncoupling were detected in *mrc1Δ* cells, but not *tof1Δ* cells. These data further support the notion that the replication disruption in long replicons resulting from loss of Mrc1 is quantitatively higher than loss of Tof1 function.

As part of the FPC, Mrc1 and Tof1 are required both for replication checkpoint signaling and, separately, for rapid and stable replication elongation (40, 41, 58–60). Previously we have characterized a truncation of Tof1, *tof1 627*, that maintains checkpoint signaling in response to hydroxyurea, but is defective for interaction with Csm3 and Csm3 linked functions (22). If replication checkpoint signaling was primarily required to prevent FPC-linked DNA damage in long replicons, we would predict that expression of *tof1 627* would suppress DNA damage accumulation. However, cells expressing *tof1 627* showed very similar accumulations of H2AP to *tof1Δ* cells (*SI Appendix*, Fig. S6*C*, (49)). Therefore, restoration of replication checkpoint signaling did not detectably rescue DNA damage in long replicons, arguing that the damage is due to the loss of the rapid and stable replisome supported by all FPC factors including Csm3.

In summary, these data show that while FPC activity promotes DNA topological stress related DNA damage in topologically constrained chromosomal contexts, FPC functions are also essential for the faithful duplication of long replicons, demonstrating two opposing sides of FPC function in cells.

## Discussion

Minimizing replication stress is essential for maintaining genome stability. Here, we show that while the FPC activity provided by Mrc1 and Tof1 is required to faithfully replicate long replicons, it also intrinsically increases DNA topological stress-linked damage in other chromosome contexts. This necessitates that cells balance out the usage of replication stress-regulating pathways according to their specific chromosomal composition and growth environment.

Ongoing DNA replication requires the constant removal of overwinding DNA topological stress (9, 11). Here, we show that two regulators of CMG helicase translocation, Mrc1/Claspin and Tof1/Timeless, determine the level of DNA topological stress acting on the fork. When these FPC factors are active, the rapidly elongating replisome generates high levels of DNA topological stress at the fork, leading to increased replication stress in regions where DNA topological stress accumulates. This illustrates a previously undescribed factor in determining the level of DNA topological stress during DNA synthesis, the rate of DNA unwinding by the CMG. Our data link the low rates of unwinding in *mrc1Δ* cells to reduced replication-linked DNA topological stress and its associated DNA damage. We also show that recruitment of Top1 to the fork by Tof1 minimizes the consequential fork stalling caused by FPC-generated DNA topological stress.

With our methods we currently observe Top1 mediated suppression of replication stress in two budding yeast chromosomal contexts known to be acutely sensitive to accumulated DNA topological stress, the centromeres, and the rDNA repeats (16). Between these two domains, the effect of DNA topological stress is stronger at the rDNA than at centromeres. We previously observed a similar difference in magnitude of DNA damage changes in cells depleted of Top2 during S phase (16). We speculate that while both centromeres and the rDNA repeats contain barriers to DNA topological stress diffusion, they differ in the extent of DNA topological stress that accumulates due to the barriers. In this model, the higher levels of transcription across the rDNA lead to higher levels of topological stress than around centromeres. Outside these two domains, DNA topological stress is reported to accumulate in other chromosomal contexts including nuclear envelope attached sites (61), at cohesin loop boundary sites along chromosome arms (12), at long or highly transcribed genes (11, 62) and other stable protein–DNA binding contexts that impede DNA duplex rotation (19). However, we do not yet observe loss of H2AP at all these sites when FPC activity is ablated or increases in H2AP following the specific loss of recruitment of Top1 to the fork. Potentially this could be due to the lack of sensitivity of our DNA damage detection methods. Alternatively, other topologically constrained chromosomal contexts could have replication-independent pathways of topoisomerase recruitment to alleviate accumulated DNA topological stress [for example through interaction with RNA pol II or transcription factors (63, 64)].

Our model that FPC-dependent rapid replication actively causes DNA damage in topologically constrained regions is consistent with the FPC being targeted by genome stability signaling pathways to minimize further DNA damage to the genome. Both DNA replication checkpoint kinases and stress-activated protein kinases target Mrc1 to reduce fork speed (65–67). In addition, in human cells Timeless is targeted during high levels of redox reactions in cells (68). In all these situations, the downstream effects of targeting FPC proteins will be to lower the incidence DNA topological stress–induced replication stress, minimizing further genome instability.

Although loss of FPC activity reduces DNA damage in some genomic areas, we demonstrate that lowering replication speed via loss of FPC activity leads to increased replication stress in long replicons across the genome. In mammalian cells long replicons are frequently found to be fragile sites under conditions of mild replication stress (7). Underreplication of long replicons has been proposed to lead to DNA breakage during mitosis, and the formation of 53BP1 foci in the following G1 phase (69). However, in our analysis the H2AP generated in long replicons following loss of FPC function is primarily detected in replicating and G2/M cells, not in G1. This indicates that the DNA lesions within long replicons are generated in S phase and not during passage through mitosis. At present, the nature of the S phase DNA lesions generated in long replicons following loss of FPC is unclear. Since Top1 recruitment by Tof1 is not required to prevent damage in long replicons these problems do not appear to be influenced by DNA topological stress. We envisage two scenarios whereby loss of FPC function results in DNA damage in long replicons. First, loss of FPC function could result in a replisome that becomes increasing unstable the further replication progresses. This would lead to strongly elevated frequencies of helicase-polymerases uncoupling at longer distances. Second, the slow replisome generated by loss of the FPC would result in more of the genome still being replicated as mitotic kinase activity rapidly increases as cells prepare for M phase. Increased mitotic kinase activity could destabilize the replisome through activities that alter the conformation of the chromatin template, for example through condensin activity (70), or could cause the activation of mitotically regulated nucleases capable of processing late replicating fork (71). Alternatively, normally dormant origins would be fired in unreplicated regions in late S phase. The replication forks generated from these late firing origins could be inherently more unstable in the absence of FPC activity.

Tof1 and Mrc1 are not equally important in preventing replication stress at long replicons. Loss of Tof1 leads to an S phase–focused increase in H2AP whereas loss of Mrc1 leads to a higher enrichment of H2AP in long replicons which persists into M phase, while also causing extensive exposure of ssDNA in these regions. These observations argue that the primary role of Tof1-Csm3 in preventing replication stress in long replicons is to stabilize and correctly orient Mrc1 in a manner that promotes rapid and stable DNA replication. Tof1-Csm3 are required for strong association of Mrc1 with the replisome, Mrc1 is not required for the interaction of Tof1-Csm3 with the replisome (29). In vitro, omission of Mrc1 from replication reactions causes greater loss in replication speed than omission of Tof1-Csm3 (41). The higher levels and persistence of DNA damage in *mrc1Δ* compared to *tof1Δ* would be consistent with replication forks persisting longer into the cell cycle where they could be subjected to deleterious processing. We cannot discount the possibility that the additional DNA damage and ssDNA exposure caused in *mrc1Δ* relative to *tof1Δ* cells could be due to checkpoint-related functions of Mrc1, for example through Mrc1 preventing the resection of stalled forks (72).

Previously Tof1 has been shown to have a prominent role in fork pausing at fork impeding protein–DNA sites. Surprisingly, we found that loss of Tof1 function and fork pausing does not lead to increased levels of DNA damage at tRNAs, centromeres, or the rDNA RFB, despite our findings via TrAEL-SEQ that Tof1 globally promotes fork pausing at these sites. This raises the question as to why does Tof1 activity enforce fork pausing at these loci if not to prevent DNA damage? We postulate that pausing at these sites is simply a consequence of Tof1-Csm3 stabilizing a rapid translocation competent replisome conformation that is inefficient at rapid bypass of stable protein–DNA complexes.

In summary, our data indicate that the rapidly replicating replisome supported by the FPC is essential for the timely and faithful replication of the genome. However, by virtue of the high levels of DNA topological stress generated by rapid replication, the FPC-supported replisome increases the frequency of fork stalling in topologically constrained chromosomal contexts. This necessitates the active recruitment of topoisomerase to the fork by Tof1 to minimize replication stress in those loci while maintaining genome stability in long replicons. We anticipate that the cellular mechanisms to resolve this tension will vary across different cell types and chromosome contexts. Our findings demonstrate a previously unappreciated cause of the genome instability observed in fast replicating cells (51) and highlight how maintaining cellular genome stability requires balancing rapid genome duplication with DNA topological stress–induced replication disruption.

## Materials and Methods

### Yeast Strains.

Strains are listed in *SI Appendix*, Table S1.

### Media and Cell Cycle Synchronization.

For exponential ChIP-SEQ experiments in glucose, cells were grown at 25 °C in YP media with 40 mg/L adenine +2% glucose to mid-log phase (~10^7^ cells/mL).

For exponential ChIP-SEQ experiments in galactose, cells were grown to ~0.7 × 10^7^ cells/mL at 25 °C in YP media with 40 mg/L adenine +2% raffinose first, then 2% galactose was added, and cells were further incubated to reach ~10^7^ cells/mL before collection.

For exponential experiments for TrAEL-SEQ, cells were grown at 30 °C in YP media with 40 mg/L adenine +2% glucose to mid-log phase (~10^7^ cells/mL).

Cell synchronizations and analysis were performed as described previously (19). See detailed protocol in *SI Appendix*.

### Flow Cytometry Analysis (FACS).

FACS was performed as described in ref. 19. See detailed protocol in *SI Appendix*. Data for FACS analysis are shown in *SI Appendix*, Fig. S7.

### ChIP-SEQ and ChIP-SEQ Data analysis.

ChIP-SEQ experiments were performed as described previously (16). Antibodies used were; H2A 1:500 (active motif), 1.6 μg/mL H2AP (Abcam), or RFA1 antibody (1:10,000, Agrisera). NGS library was prepared using the NEBnext Ultra II library kit. See detailed protocol in *SI Appendix*.

Data analysis for ChIP-SEQ was performed as described previously (16). Briefly, Illumina basespace (https://basespace.illumina.com/home/index) was used to generate FASTQ files. H2A and H2AP sequences were aligned without trimming to a reference genome (R64-1-1, *S. c.* S288c assembly from Saccharomyces Genome Database) using Bowtie 2 (https://bowtie-bio.sourceforge.net/bowtie2/index.shtml). RFA1 reads were aligned to the same reference genome but the LTR-retrotransposons were masked. SAM files were then converted into sorted BAM files by using SAMtools (http://samtools.sourceforge.net/).

For RFA1 analysis duplicates were removed using picard (https://broadinstitute.github.io/picard) and the resulting BAM files were used for Model-based Analysis of ChIP-SEQ by MACS2 (https://github.com/macs3-project/MACS/wiki/Install-macs2), using the “call peak” function to generate genome-wide score data. Enrichment tracks were then extracted by the bdgcmp function. The data were sorted into 50 bp bins and normalized to have a mean value of 1. Moving average (bin number indicated at each figure) was used to smooth the data which was used for meta data analysis and plotting using custom-made R programs.

### Relative Copy Number Determination.

Libraries for relative copy number determination were prepared as described for the input preparation for RFA1-ChIP. Reads were aligned LTR-retrotransposon masked reference genome, duplicates were removed using picard, and reads were summed to 50 bp bins using sam- to bincount program (https://github.com/yasukasu/sam-to-bincount) described in ref. 73. Read per million values were calculated (rDNA values ignored) and values from forward and reverse strands were summed using custom R scripts.

### TrAEL-SEQ.

TrAEL-SEQ experiments were performed as described earlier in ref. 54.

### TrAEL-SEQ Data Analysis.

UMI deduplicated mapped reads from TrAEL-SEQ experiments were generated as described in ref. 54. Mapped reads were then analyzed using SeqMonk v1.47 (https://www.bioinformatics.babraham.ac.uk/projects/seqmonk/). Minimum mapping quality of one was applied, and reads were truncated to one nucleotide at the 5′ end. Running windows of probe size 10 bp and step size 10 bp were generated and the reads were exported to bedgraph file. Custom-made R programs were then used to calculate reads per million values (reads around rDNA were ignored). Reads per million values were then smoothed by moving averages indicated at each figure for plotting using custom-made R programs. When plotting metadata CUP1 region (±5 kb) was ignored.

For plotting TrAEL-SEQ data at rDNA regions, values were normalized to relative copy numbers over the rDNA region. Ratio of the mean of relative copy numbers from positions 450 kb to 470 kb on chromosome XII (rDNA region) from *tof1Δ* and *wt* cells was used as a correction factor for the normalization.

Read polarity was calculated as follows: read polarity = (F − R)/(F + R), where F is TrAEL-SEQ reads per million values on the forward strand and R is TrAEL-SEQ reads per million values on the reverse strand.

### DNA Preparation, Gel Electrophoresis, and Southern Blotting for Plasmid Catenation.

DNA preparation, agarose gel electrophoresis, and Southern blotting were carried out as described in ref. 74. See detailed protocol in *SI Appendix*. Analysis was carried out as described previously (19). The densitometry analysis of individual blots is shown in Dataset S1. Box plots were generated using BoxplotR (http://shiny.chemgrid.org/boxplotr/).

## Supplementary Material

Appendix 01 (PDF)

Dataset S01 (XLSX)

## Data Availability

Processed sequencing data were deposited in GEO submission number (49) GSE239967.
