# Supplementary material for: The fork protection complex generates DNA topological stress–induced DNA damage while ensuring full and faithful genome duplication
Source: Proc Natl Acad Sci U S A. 2024 Nov 26;121(49):e2413631121. doi: 10.1073/pnas.2413631121 (PMC11626154; doi:10.1073/pnas.2413631121)
Supplement: Supplementary file 1 — Appendix 01 (PDF) [file pnas.2413631121.sapp.pdf]

PNAS

Supporting Information for

The Fork Protection Complex generates DNA topological stress induced DNA damage while ensuring full and faithful genome duplication.

Andrea Keszthelyi, Sahar Mansoubi, Alex Whale, Jon Houseley and Jonathan Baxter*

*corresponding author Jonathan Baxter
Email: Jon.Baxter@sussex.ac.uk;

This PDF file includes:

Supporting text
Figures S1 to S7
Tables S1 to S2
SI References

Other supporting materials for this manuscript include the following:

Datasets S1

Supporting Text

Supporting Information (SI) for Methods.

Yeast strains

Strains were derived from the W303 background *(ade2–1 ura3–1 his3–11, trp1–1 leu2–3, can1–100*) as listed in Table S1.

Media and Cell Cycle Synchronization

Cell synchronizations were performed as described previously (1). Briefly cultures were grown in YP media with 40 ml/l adenine + 2% raffinose to log phase, then 10 µg/ml alpha factor (Genscript) was added. 2% of galactose was added when ~90% of cells were unbudded (at ~120 min). 20 minutes after galactose addition 50 µg/ml doxycycline (Sigma-Aldrich) and 5 µg/ml additional alpha factor was added. 10 minutes after this the temperature was shifted to 37°C for 1h. Cells were washed three times with YP + 40 mg/l adenine + 2% raffinose + 2% galactose 50 µg/ml doxycycline and resuspended in the same media to release them from G1 block. For mitotic arrested samples, nocodozole (Sigma-Aldrich) was added to cultures at 10 µg/ml 45 minutes after time 0 min (addition of the first wash), and cells were collected at 95 min. For G1 arrested samples, 10 µg/ml alpha factor was added after 70 min and cells were collected at 160 min. Cell cycle phases were confirmed by Flow cytometry analysis (FACS)(Figure S7).

Flow cytometry analysis (FACS)

FACS was performed as described in (1). 500 µl of culture was collected by centrifugation and fixed with 70% ethanol. Cells were then RNAse treated in 1 ml of 50 mM Tris-HCl pH8 with 5 mg/ml RNaseA (Sigma-Aldrich) at 37°C overnight, followed by protease treatment in 1 ml 5 mg/ml pepsin (Sigma-Aldrich) and 5 µl/ml concentrated HCl at 37°C for 30 minutes. After washing with 50 mM Tris-HCl pH8 cells were resuspended in 50 mM Tris-HCl pH8 with 0.5 mg/ml Propidium iodide (Sigma-Aldrich) and sonicated briefly. FACS was performed using BD Accuri C6 sampler. Analysis was carried out using FCS express 4 flow software. Data for FACS analysis is shown in Figure S7.

ChIP-SEQ

For ChIP-SEQ experiments were performed as described previously (2). Starting volume was 25 ml from the cultures per each antibody used. Cells were washed and resuspended in YP media, and fixed by adding 1% formaldehyde (Sigma-Aldrich) followed by 45 min incubation at 25°C. Formaldehyde was quenched by addition of glycine (Alfa Aesar, 125 mM final concentration, 5 min incubation). Cells were then washed with cold PBS, pelleted and frozen in liquid nitrogen. For a subset of experiments (see Table S2) *S. p.* cells containing the HO-endonuclease inducible system (AW507) were added to allow normalization as described in (2).

Frozen pellets were resuspended in 500 µl SDS buffer (1% SDS, 10 mM EDTA, 5M Tris HCl, cOmplete Tablets, Mini EDTA-free EASYpack (Roche), PhosSTOP (Roche)). 200 µl of 0.5 mm silica beads were added to the samples and cells were lysed in a FASTPREP machine (5 rounds of 1 min at 6.5 power). Silica beads were then separated and discarded and IP buffer (0.1% SDS, 1.1% Triton-X-100, 1.2 mM EDTA, 16.7 mM TRIS HCl (pH8), cOmplete Tablets, Mini EDTA-free EASYpack (Roche), PhosSTOP (Roche)) was added to the lysate to a final volume of 1 ml. Samples were then sonicated by Focused-Ultrasonicator (Covaris, M220, Average incident power – 7.5 Watts, Peak Incident Power – 75

Watts, Duty Factor – 10 %, Cycles/Burst – 200, Duration – 20 min). Supernatant was diluted to 1/10 of the volume of the starting culture (e.g. started from 50 ml diluted to 5 ml). 1/1000 of the starting volume of protein A Dynabeads (Invitrogen) and protein G Dynabeads (Invitrogen) each were mixed (e.g. 50-50 ul if 50 ml starting culture was used). Beads were washed 3 times in IP buffer then added to the samples followed by 2 h incubation at 4°C. Beads were separated and discarded and 2-2 ml of the supernatant was incubated with either H2A 1:500 (active motif), 1.6 µg/ml H2AP (Abcam) or RFA1 antibody (1:10000, Agrisera) on a rotating wheel at 4°C for 15 – 20 h. The rest of the sample was kept for input. 30-30 µl of protein A and G Dynabeads were washed 3x with IP buffer and added to each antibody reaction and incubated at 4°C for 4 h. Beads were then washed at 4°C for 6 min in TSE-150 (1% Triton-X-100, 0.1% SDS, 2 mM EDTA, 20 mM Tris HCl (pH8), 150 mM NaCl), followed by TSE-500 (1% Triton-X-100, 0.1% SDS, 2 mM EDTA, 20 mM TrisHCl (pH8), 500 mM NaCl), followed by LiCl wash (0.25 M LiCl, 1% NP-40, 1% dioxycholate, 1 mM EDTA, 10 mM Tris HCl (pH8)) and finally Tris-EDTA (TE pH8). Samples were then eluted in 400 µl elution buffer (1% SDS, 0.1 M Na HCO_3), for 30 min at room temperature. For input samples 50 µl starting material was mixed with

150 µl of elution buffer. Pull-down and input samples were reverse crosslinked, and protease treated by adding NaCl (500 mM final concentration) and proteinase K (Invitrogen, 500 µg/ml final concentration) followed by incubation at 65°C overnight. Samples were then RNase treated (25 µg/ml final concentration of DNase-free RNase (Roche) at 37°C for 30 min). DNA was then purified with Qiagen PCR purification kit. NGS libraries were prepared using NEBnext Ultra II library kit (NEB). For H2A and H2AP ChIP-seq experiments 13 cycles were used for PCR enrichment. DNA was size selected and purified using AMPure XP beads. DNA yield was measured by Qubit 2.0 Fluorometer (Life technologies). For input and RFA1 libraries 34 µl from the RFA1 IP samples and 1 ng DNA in 34 ul water from the input was used. Complementary strand for the ssDNA was synthetised by first adding 5 µl 10 x NEB2.1 buffer and 5 µl of random primers (8N, 3 mg/ml stock). Samples were then boiled at 95°C for 5 minutes and immediately placed to ice for 5 minutes. 5 µl 10 x dNTP with dUTP instead of dTTP (2 mM each) and 1 µl T4 polymerase (NEB) were then added followed by incubation at 37°C in a thermal cycler for 20 min. 5 µl 0.5 M EDTA (pH 8) was immediately added to stop the reaction. This was then used to generate libraries using Ultra II library kit (NEB) with 16 cycles for PCR enrichment. Paired end sequencing was performed using NextSeq 500 (42 bp reads from each side) system.

ChIP-SEQ data analysis

Data analysis for ChIP-SEQ was performed as described previously (2). Briefly, Illumina basespace (https://basespace.illumina.com/home/index) was used to generate FASTQ files. H2A and H2AP sequences were aligned without trimming to a reference genome (R64-1-1, *S. c.* S288c assembly from Saccharomyces Genome Database) using Bowtie 2 (http://bowtie bio.sourceforge.net/bowtie2/index.shtml). RFA1 reads were aligned to the same reference genome but the LTR-retrotransposons were masked. SAM files were then converted into sorted BAM files by using SAMtools (http://samtools.sourceforge.net/).

For RFA1 analysis duplicates were removed using picard (https://broadinstitute.github.io/picard) and the resulting BAM files were used for Model-based Analysis of ChIP-SEQ (MACS2), using the ‘call peak’ function to generate genome wide score data. Enrichment tracks were then extracted by the bdgcmp function. The data was sorted into 50 bp bins and normalized to have a mean value of 1.

Moving average (bin number indicated at each figure) was used to smooth the data which was used for meta data analysis and plotting using custom made R programs.

Relative copy number determination

Libraries for relative copy number determination were prepared as described for the input preparation for RFA1-ChIP. Reads were aligned LTR-retrotransposon masked reference genome, duplicates were removed using picard, and reads were summed to 50 bp bins using sam- to bincount program (https://github.com/yasukasu/sam-to-bincount) described in (3). Read per million values were calculated (rDNA values ignored) and values from forward and reverse strands were summed using custom R scripts**.**

TrAEL-SEQ

TrAEL-SEQ experiments were performed as described earlier in (4).

TrAEL-SEQ data analysis

UMI deduplicated mapped reads from TrAEL-SEQ experiments were generated as described in (4) . Mapped reads were then analysed using SeqMonk v1.47 (https://www.bioinformatics.babraham.ac.uk/projects/seqmonk/). Minimum mapping quality of 1 was applied, and reads were truncated to 1 nucleotide at the 5' end. Running windows of probe size 10 bp and step size 10 bp were generated and the reads were exported to bedgraph file. Custom made R programs were then used to calculate reads per million values (reads around rDNA were ignored). Reads per million values were then smoothed by moving averages indicated at each figure for plotting using custom made R programs. When plotting metadata CUP1 region (+-5kb) was ignored.

For plotting TrAEL-SEQ data at rDNA regions, values were normalised to relative copy numbers over the rDNA region. Ratio of the mean of relative copy numbers from positions 450 kb – 470 kb on chromosome XII (rDNA region) from *tof1Δ* and *wt* cells was used as a correction factor for the normalisation.

Read polarity was calculated as: read polarity = (F − R)/(F + R), where F is TrAEL-SEQ reads per million values on the forward strand and R is TrAEL-SEQ reads per million values on the reverse strand.

DNA preparation for gel electrophoresis

Frozen pellets were re-suspended in lysis buffer (50mM Tris-HCl pH 8.0, 100mM NaCl, 10mM EDTA, 1%SDS) and the cell wall removed by incubation with 80 units/ml Lyticase (Sigma-Aldrich) and 1% β-mercaptoethanol (Sigma-Aldrich) at 37°C for 5 minutes. DNA was then extracted with phenol/chloroform/isoamylalcohol (25:24:1) and the aqueous layer removed using phase lock tubes (Scientific Laboratory Supplies). DNA was precipitated with 2 volumes of 100% ethanol and washed with 70 % ethanol before being re-solubilized in 10mM Tris pH8.0.

Gel electrophoresis for plasmid catenation

For catenation 2D gels the DNA was nicked with either Nb.BsmI or Nb.BsrDI (NEB) according to the manufacturer's instructions. Nicked catenanes were separated in the first dimension on a 0.4% agarose (Megasieve, Flowgen) gel in 1x TBE (Tris-base, Boric Acid, EDTA) at 1.2V/cm for 13-17h at room temperature. The respective lanes were excised and embedded into a 0.8-1.2% (depending on plasmid size) agarose (Megasieve, Flowgen) gel and run at 2-4.8V/cm in 1x TBE (at 4°C if more than 2V/cm were used).

Southern blotting

Non-radioactive Southern blotting and detection were carried out as described in (5): the gel was washed in depurination buffer (0.125 M HCl), denaturation buffer (0.5 M NaOH, 1.5 M NaCl) and neutralization buffer (0.5 M Tris-HCl, 1.5 M NaCl pH 7.5). DNA was transferred onto Hybond-N+ membrane (GE Healthcare) by capillary action in 20X SCC (NaCl, Trisodium citrate, pH 7). After transfer, DNA was ultraviolet cross-linked to the membrane using a UV Stratalinker 1800 (Stratagen) at 1200 J/m. The membrane was blocked at 60°C (5X SSC, 5% Dextran Sulphate (Sigma-Aldrich), 0.2% Tropix I-Block (Applied Biosystems), 0.1% SDS). Plasmid DNA was probed with DNA amplified from sequences of pRS316. Labelling and detection used random prime labelling module incorporating fluorescein tagged dUTP (Roche). Washes were carried out at 60°C in 1X SSC with 0.1% SDS, followed by 0.5X SSC and 0.1% SDS. The membrane was blocked in AB buffer (100 mM Tris-HCl, 150 mM NaCl (pH 7.5)) with 1% milk. Hybridized fluorescein tagged dUTP was detected with alkaline phosphatase Anti-fluorescein-AP Fab fragments (Roche) followed by washing in AB buffer + 0.2% Tween 20 (Sigma-Aldrich) and revealed with CDP-Star (GE Healthcare). Non-saturating exposures

acquired on an ImageQuant LAS4000 system (GE Healthcare). Densitometry analysis was carried out using ImageQuant TL software. Overexposed images were taken to clearly identify the CatAn=1 signal, which was often weak in non-saturating exposures. Analysis was carried out as described previously (1). The densitometry analysis of individual blots is shown in Dataset S1. Box plots were generated using BoxplotR (http://shiny.chemgrid.org/boxplotr/).

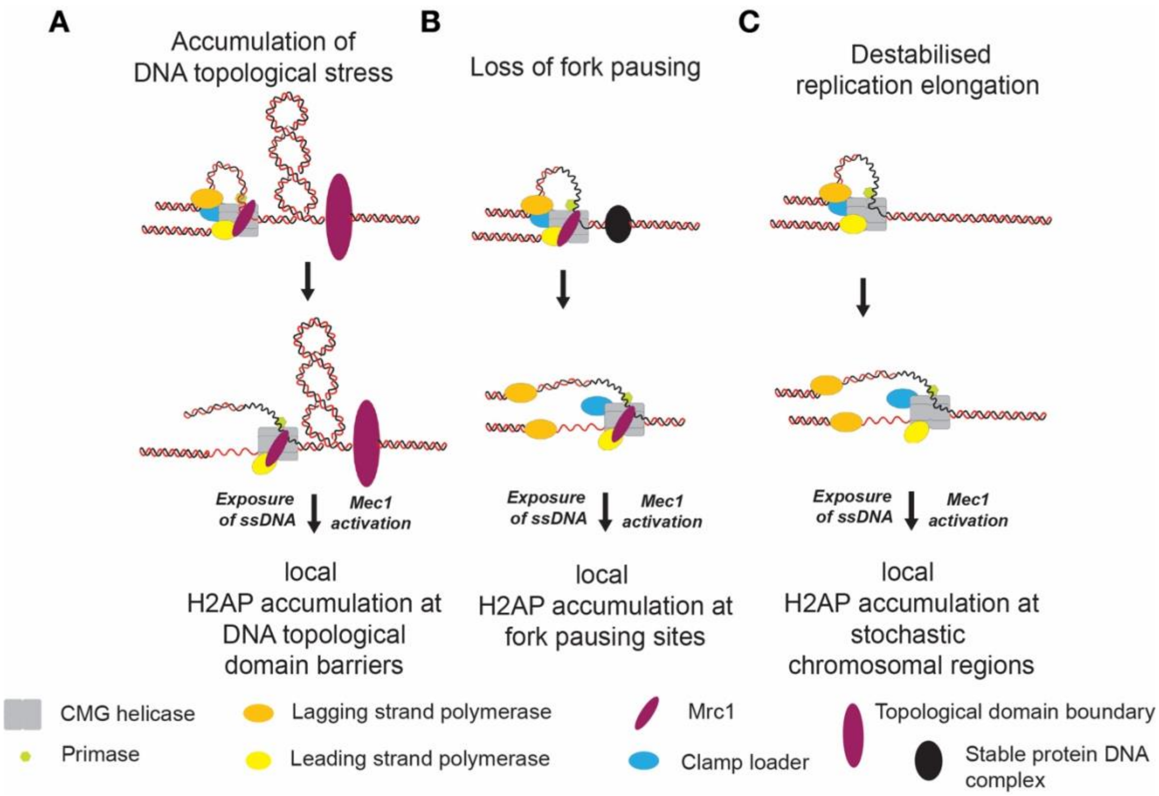

Fig. S1. Models of types of replication stress caused by loss of *TOF1*

(A) In regions containing DNA topological boundary complexes, the DNA topological stress generated by DNA unwinding cannot diffuse away from the site of generation, and will accumulate locally. In the absence of Tof1, Top1 recruitment is reduced causing the stress to accumulates to a level where replication stalling occurs. This leads to fork collapse and exposure of nascent strands to nuclease processing.

(B) At sites of stable protein-DNA complexes, DNA replication is paused in a Tof1 dependent manner. Loss of Tof1 could allow deleteriously rapid passage of the replication fork through the DNA bound protein barrier. This could lead to helicase-polymerase uncoupling and exposure of nascent strands to nuclease processing.

(C) In the absence of FPC function (from loss of Tof1 or Mrc1) the fork becomes subject to more frequent stochastic helicase-polymerase uncoupling due to the inherent instability of the replisome. Uncoupling will lead to exposure of nascent strands which could then become subject to nuclease processing.

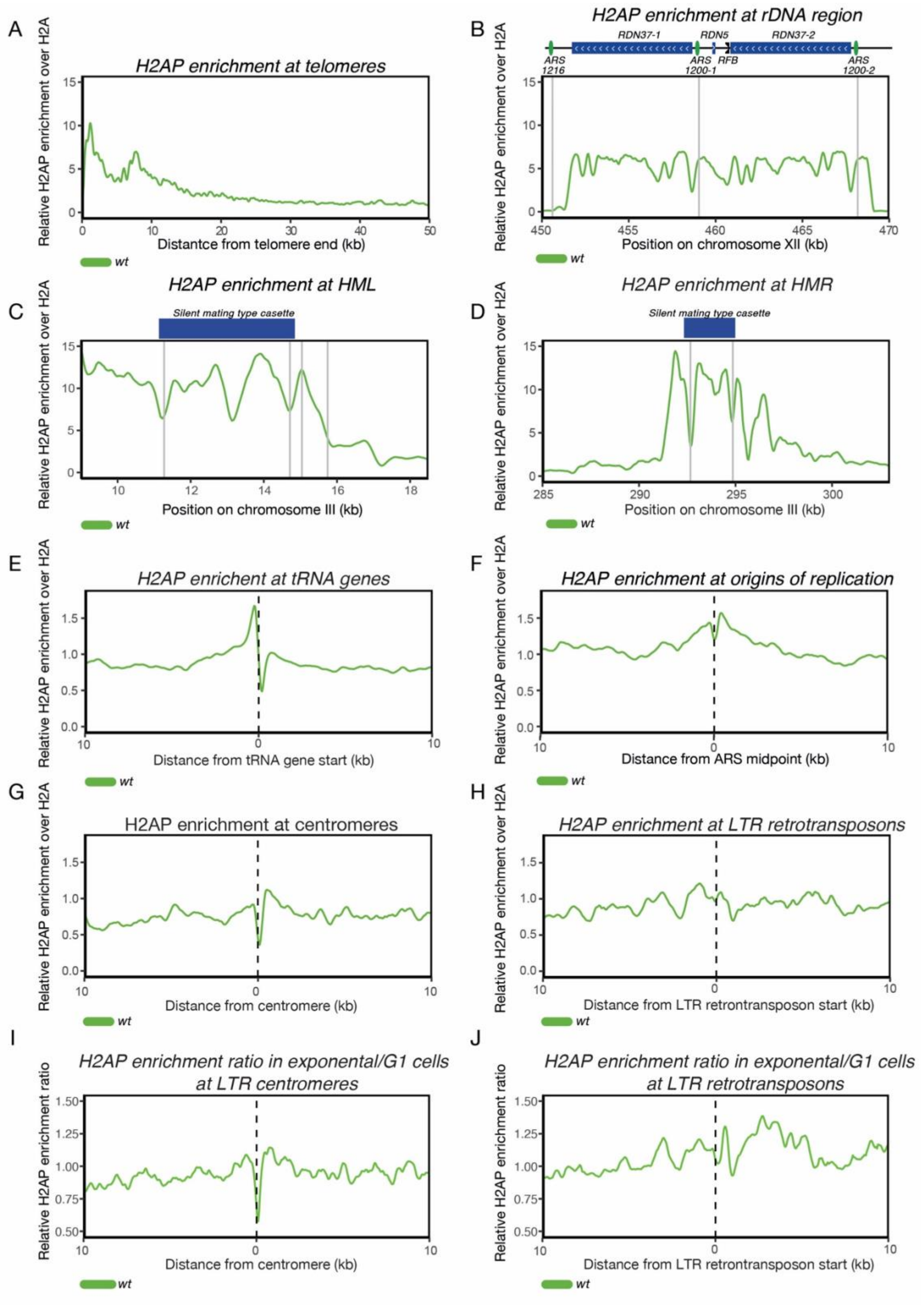

Fig. S2. H2AP enrichment signal is increased in *wt* cells at specific regions

Relative H2AP enrichment in wildtype cells at (A) telomeres, (B) rDNA repeats, (C) HML - silent mating type locus, (D) HMR - silent mating type locus (grey vertical lines indicate positions of ARS sequences in the HML and HMR regions), (E) upstream and downstream regions of tRNA, (F) origins of replication (ARS sequences), (G) centromeres and (H) LTR sequences. (I, J) to illustrate changes in H2AP linked to passage through S phase we plotted the ratio of H2AP detected in exponential cells to that detected in G1 arrested cells for (I) centromeres and (J) LTR sequences.

Smoothing with moving average over 7 bins (50 bp bin size) was applied.

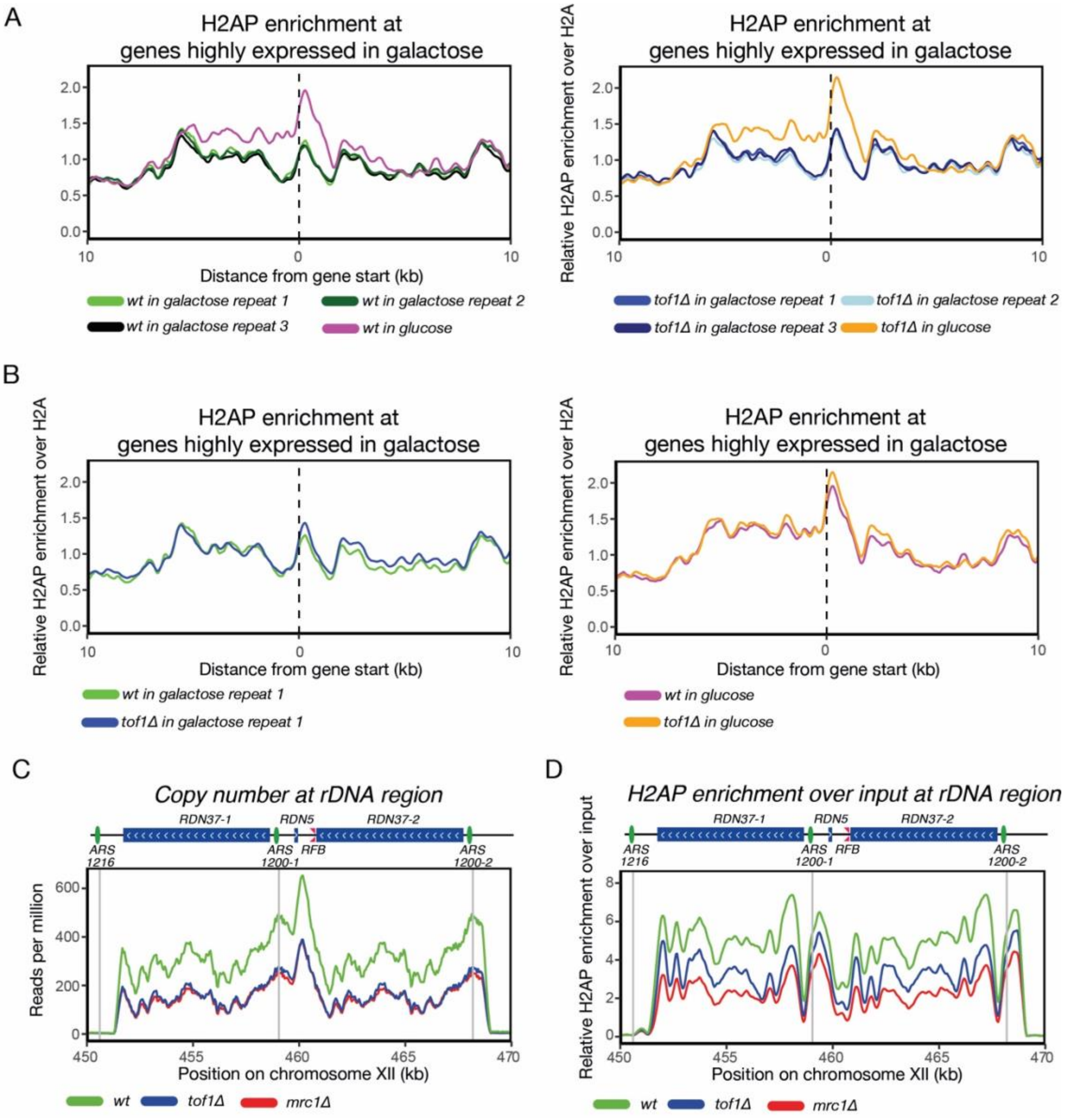

Fig. S3. Repression of galactose inducible gene in glucose media causes H2AP accumulation both in wt and TOF1 deleted cells. H2A enrichment at origins of replications is not affected by loss of Tof1. H2AP enrichment at rDNA is not dependent on copy number

Determination of highly expressed genes in galactose was based on the dataset in (6) expression in YP galactose vs reference pool >1.5. (A) Relative H2AP enrichment over H2A at genes highly expressed in galactose in *wt* (left) and *tof1*Δ (right) grown in media containing galactose or glucose. (B) Relative H2AP enrichment over H2A at genes highly expressed in galactose in *wt* and *tof1*Δ cells grown in media containing galactose (left) or glucose (right). (C) Copy number across rDNA region. (D) The relative enrichment of H2AP over input ChIP across the rDNA repeats in *wt* and *tof1*Δ cells. Grey vertical lines indicate positions of ARS sequences in the region. Smoothing with moving average over 7 bins (350bp) was applied. Grey vertical lines indicate positions of ARS sequences in the region. Smoothing with moving average over 7 bins (50bp bin) was applied

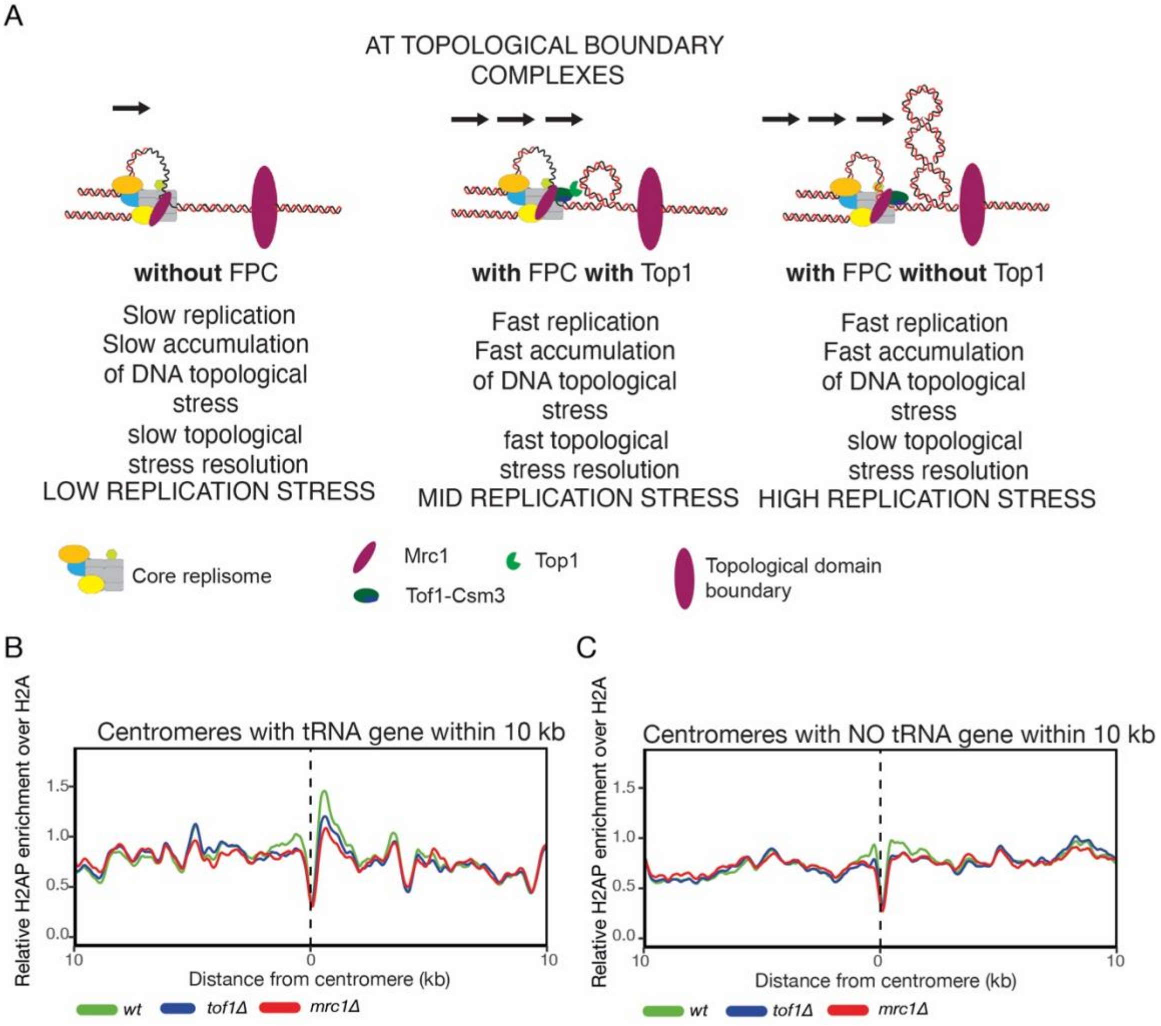

Fig. S4. Model for how FPC activities regulate DNA topological stress at topological boundary complexes such as centromeres and the rDNA, and how tRNA genes around centromeres alter DNA topological stress accumulation

(A) Middle panel - In regions containing DNA topological boundary complexes the DNA topological stress generated by DNA unwinding cannot diffuse away from the site of generation and will accumulate locally, potentially stalling replication. - left hand panel - In the absence of an intact FPC, DNA unwinding is slow and therefore DNA topological stress accumulation is slow. Therefore, diffusing topoisomerases are sufficient to prevent stress accumulating to the point where it could stall replication. - right hand panel - However, if the FPC is intact, replication is fast, DNA unwinding is fast and therefore DNA topological stress accumulates quickly. Without active Top1 recruitment the stress accumulates to a level where replication frequently stalls.

(B, C) Relative H2AP enrichment over H2A in *wt, tof1*Δ *and mrc1*Δ cells at (B) around centromeres with tRNA within 10kb of the centromere (C) around centromeres without tRNA within 10kb of the centromere.

Smoothing with moving average over 7 bins (50bp bin size) was applied.

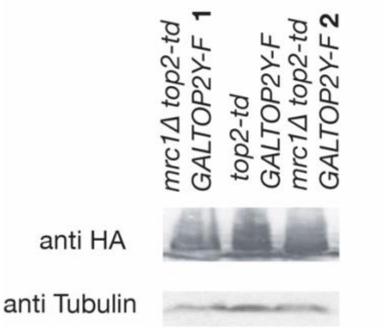

Fig. S5. Additional information for Loss of Mrc1 reduces the level of DNA topological stress ahead of the replication fork during replication of plasmid *3xtRNApRS316* in *GAL1-top2Y-F top2td pRS316.*

Western blot of HA tagged Top2Y-F protein expressed when grown in 2% galactose in both *GAL1-top2Y-F top2td pRS316* and two individual isolates of *mrc1Δ GAL1-top2Y-F top2td pRS316.*

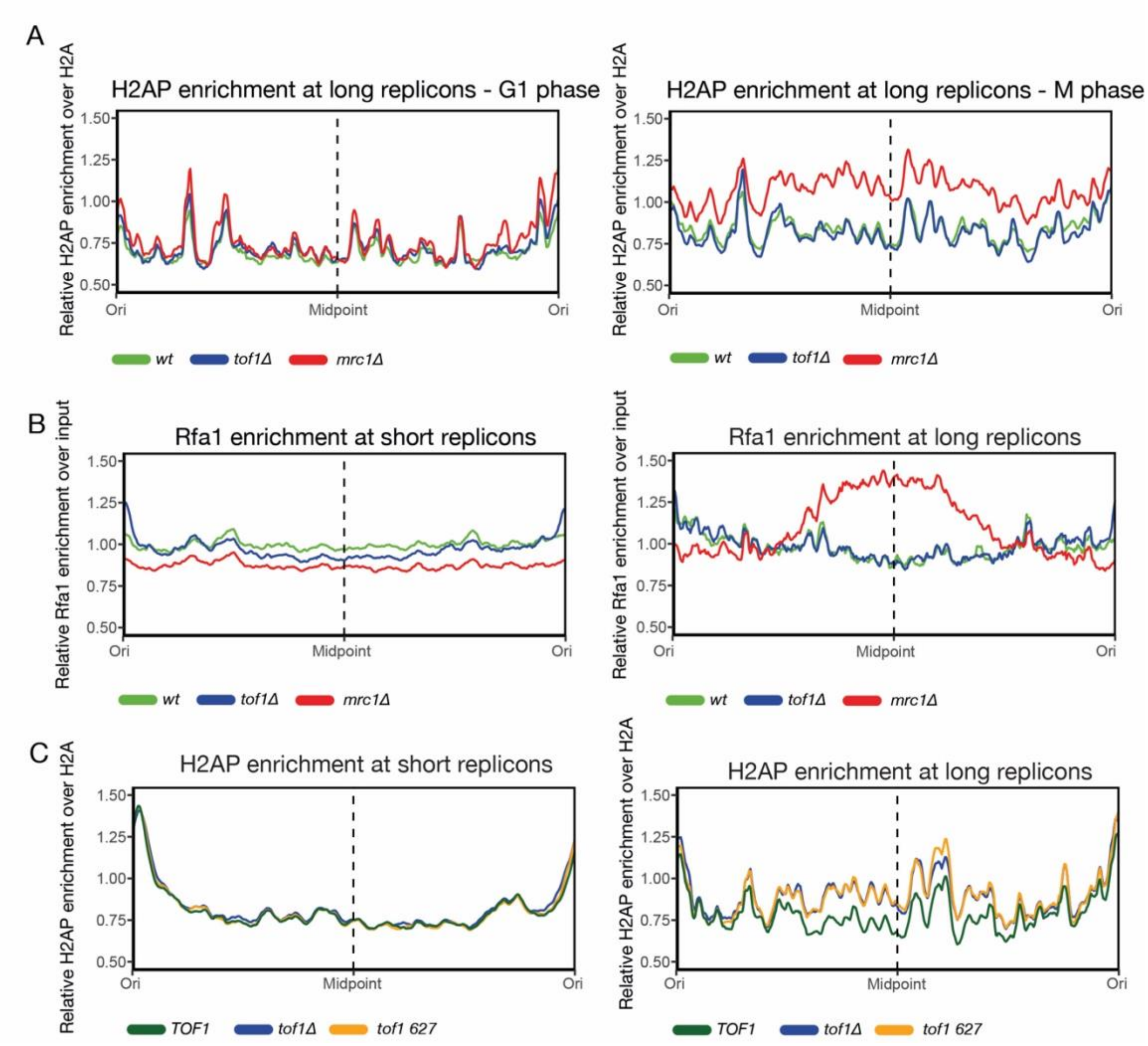

Fig. S6. Persistent DNA damage and ssDNA is focused in long replicons in *mrc1*∆ cells.

(A) Relative H2AP enrichment over H2A in *wt, tof1*∆ *and mrc1*∆ cells over long replicons (>60kb) in G1 synchronized (treated with alpha factor) (left) and mitotic cells (arrested with nocodazole) (right). Smoothing with moving average over 20 bins (50bp bin size) was applied. Correct cell cycle synchronization of all cultures processed for (A) was confirmed by FACS for DNA content – (Figure S7).

(B) Relative Rfa1 enrichment over input in *wt, tof1*∆ *and mrc1*∆ cells over short replicons (20kb to 50kb) (left) and long replicons (>60kb) (right) in exponential cells. Smoothing with moving average over 20 bins (50bp bin size) was applied.

(C) Relative H2AP enrichment in *TOF1wt, tof1*∆ and *tof1 627* cells over short replicons (20kb to 50kb) (left) and long replicons (>60kb) (right). Smoothing with moving average over 20 bins (50bp bin size) was applied.

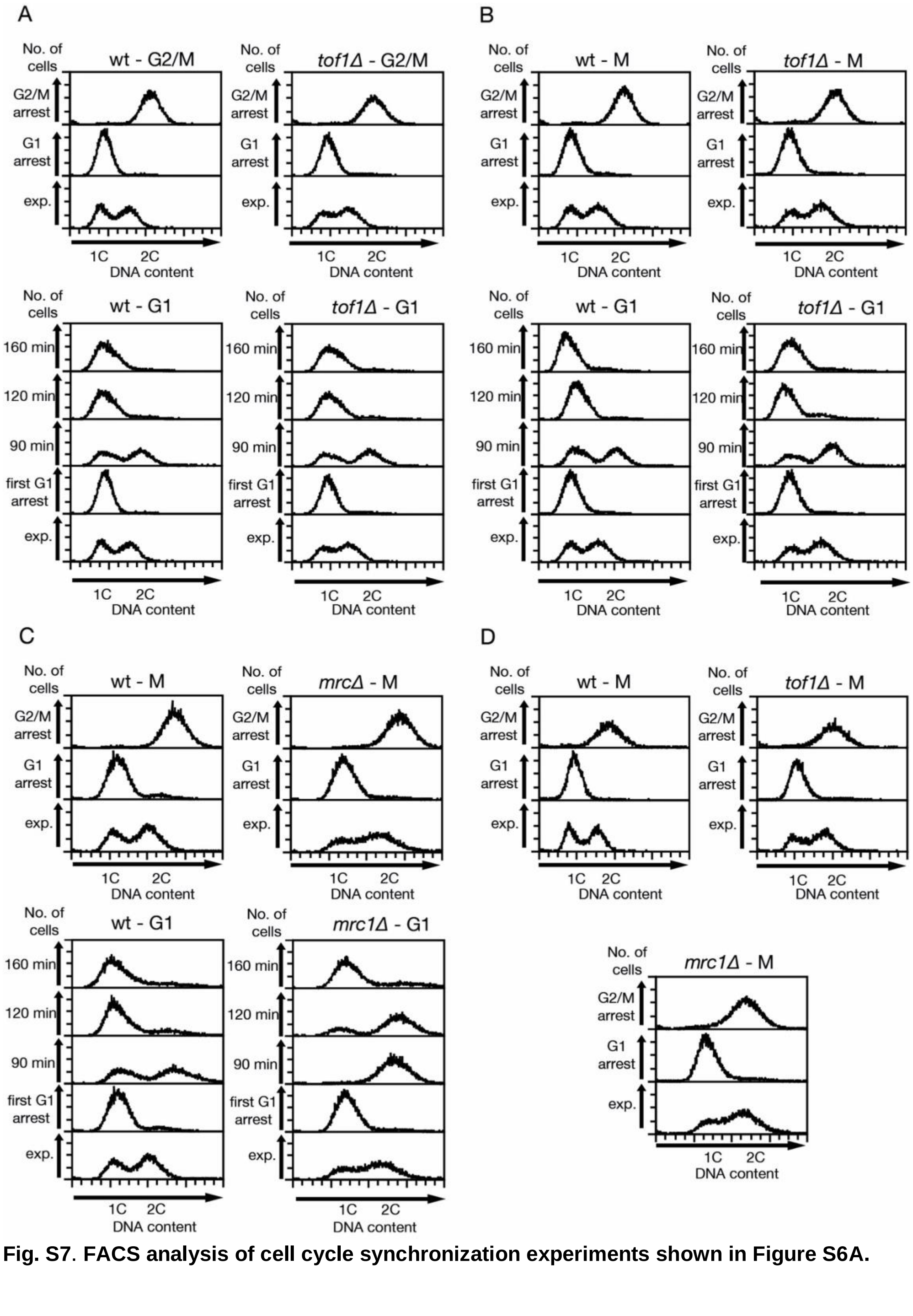

Fig. S7. FACS analysis of cell cycle synchronization experiments shown in Figure S6A.

(A) G2/M (top) and G1 (bottom) arrest *of wt* and *tof1*Δ cells.

(B) repeat of (A) used in Figure S6A. (C) G2/M (top) and G1 (bottom) arrest of *wt and mrc1*Δ cells. (D) G2/M arrest of *wt, tof1*Δ and *mrc1*Δ cells.

Table S1. Yeast strains used in this study.

Number	Name	Genotype	Source
1991	*wt*	*MATa, ade2–1 his3–11 leu2–3 trp1–1 ura3–1 can1–100, UBR1::GAL1–10-Ubiquitin-M-LacI fragment-Myc-UBR1 (HIS3), leu2–3::pCM244 (CMVp-tetR'-SSN6, LEU2) x3*	S. Tanaka, J. F. Diffley, *Nature Cell Biology*. **4**, 198–207 (2002). (7)
1993	*tof1Δ*	*1991 + tof1Δ::hphNT1*	R. Westhorpe et al. *Nucleic Acids Res.* 48(21): 12169–12187 (2020) (8)
2538	*ars517Δ*	*1991 + ars517Δ::natNT2*	This study
2551	*tof1Δ ars517Δ*	*1993 + ars517Δ::natNT2*	This study
2075	*mrc1Δ*	*1991 + mrc1Δ::hphNT1*	This study
572	*wt*	*Matα ade2-1 his3-11 leu2-3 trp1-1 ura3-1 can1-100*	Baxter laboratory
2856	*tof1Δ*	*572 + tof1Δ::hphNT1*	This study
1528	*TOF1wt*	*1991 + TOF1::TOF1-codon-optimised-wildtype (natNT2)*	R. Westhorpe et al. *Nucleic Acids Res.* 48(21): 12169–12187 (2020) (8)
1546	*tof1 997*	*1991 + TOF1::tof1-codon-optimised-997–1238Δ (natNT2)*	R. Westhorpe et al. *Nucleic Acids Res.* 48(21): 12169–12187 (2020) (8)
1549	*tof1 627*	*1991 + TOF1::tof1-codon-optimised-627–1238Δ (natNT2)*	R. Westhorpe et al. *Nucleic Acids Res.* 48(21): 12169–12187 (2020) (8)
619	*mrc1Δ top2-4 3xtRNApRS316*	*S. cerevisiae W303 background. Mat a his4-539 lys2-801 ura3-52 mrc1Δ::natNT2 top2-4 pRS316-3x tRNA*	This study
774	*GAL1-top2Y-F top2td pRS316*	*1991 + kanMX- tT A-tetO2-UB-DHFRts-myc-top2, trp1::pGAL-HA-TOP2Y-F(Y783-F)*	J. Baxter and J Diffley Mol. Cell 30, 7.90-802. (9)
778	*mrc1Δ GAL1-top2Y-F top2td pRS316*	*774 + mrc1Δ::URA3*	This study
1026	*tof1Δ top2-4 cells 3xtRNApRS316*	*S. cerevisiae W303 background. Mat a his4-539 lys2-801 ura3-52 tof1Δ::hphMX top2-4 pRS316-3x tRNA*	S. Schalbetter et al. *PNAS.* 112 (33) E4565-E4570 (2015) (1)
1136	*mrc1Δtof1Δ top2-4 3xtRNApRS316*	*1026 + mrc1Δ::natNT2*	This study

Table S2. Number of repeats and conditions used for sequencing experiments

Experiment Strain	Experimental condition (no of repeats)	No. of all repeats	Datasets used	Used in figures
H2AP ChIP exponential *wt*	H2AP ChIP-SEQ, Exponential in glucose with *S. pombe* (1)<br>H2AP ChIP-SEQ, Exponential in galactose with *S. pombe* (2)<br>H2AP ChIP-SEQ, Exponential n galacose (1)	4	wt_expglu_sp_H2AP (GSM7678361);<br>wt_expglu_sp_H2A (GSM7678362)<br>wt_exp_sp_H2AP_rep1 (GSM7678363);<br>wt_exp_sp_H2A_rep1 (GSM7678364)<br>wt_exp_sp_H2AP_rep2 (GSM7678365);<br>wt_exp_sp_H2A_rep2 (GSM7678366)<br>wt_exp_H2AP (GSM7678367);<br>wt_exp_H2A (GSM7678368)	Fig 1. A-D<br>Fig 5. F<br>Fig 6. A<br>Fig 7. A<br>Supp. Fig. 2. A-J<br>Supp. Fig. 3. A-D<br>Supp. Fig. 4. B-C
H2AP ChIP exponential *tof1*Δ	H2AP ChIP-SEQ, Exponential in glucose with *S. pombe* (1)<br>H2AP ChIP-SEQ, Exponential in galactose with *S. pombe* (2)<br>H2AP ChIP-SEQ, Exponential in galacose (1)	4	tof1_expglu_sp_H2AP (GSM7678369);<br>tof1_expglu_sp_H2A (GSM7678370)<br>tof1_exp_sp_H2AP_rep1 (GSM7678371);<br>tof1_exp_sp_H2A_rep2 (GSM7678372)<br>tof1_exp_sp_H2AP_rep2 (GSM7678373);<br>tof1_exp_sp_H2A_rep2 (GSM7678374)<br>tof1_exp_H2AP (GSM7678375);<br>tof1_exp_H2A (GSM7678376)	Fig 1. A-B<br>Fig 2. A-B<br>Fig 5. F<br>Fig 6. A<br>Fig 7. A<br>Supp. Fig. 3. A-D<br>Supp. Fig. 4. B-C
H2AP ChIP exponential *mrc1*Δ	H2AP ChIP-SEQ, Exponential in glucose with *S. pombe* (1)<br>H2AP ChIP-SEQ, Exponential in galactose with *S. pombe* (1)<br>H2AP ChIP-SEQ, Exponential in galacose (1)	3	mrc1_expglu_sp_H2AP (GSM7678385);<br>mrc1_expglu_sp_H2A (GSM7678386)<br>mrc1_exp_sp_H2AP (GSM7678387);<br>mrc1_exp_sp_H2A (GSM7678388)<br>mrc1_exp_H2AP (GSM7678389);<br>mrc1_exp_H2A (GSM7678390)	Fig 1. C.-D.<br>Fig 7. A<br>Supp. Fig. 3. C-D<br>Supp. Fig. 4. B-C
H2AP ChIP exponential *TOF1wt*	H2AP ChIP-SEQ, Exponential in galactose with *S. pombe* (1)<br>H2AP ChIP-SEQ, Exponential in galacose (1)	2	TOF1wt_exp_sp_H2AP (GSM7678437);<br>TOF1wt_exp_sp_H2A (GSM7678438);<br>TOF1wt_exp_H2AP (GSM7678439);<br>TOF1wt_exp_H2A (GSM7678440);	Fig 2. A-B<br>Supp. Fig. 6 C
H2AP ChIP exponential *tof1 997*	H2AP ChIP-SEQ, Exponential in galactose with *S. pombe* (1)<br>H2AP ChIP-SEQ, Exponential in galacose (1)	2	tof1997_exp_sp_H2AP (GSM7678441);<br>tof1997_exp_sp_H2A (GSM7678442);<br>tof1997_exp_H2AP (GSM7678443);<br>tof1997_exp_H2A (GSM7678444);	Fig 2. A-B<br>Supp. Fig. 6 C
TrAEL-SEQ, exponential *wt*	TrAEL-SEQ, Exponential in glucose (2)	2	wt_TrAEL_seq_rep1 (GSM7678449)<br>wt_TrAEL_seq_rep2 (GSM7678450)	Fig 5. A-E<br>Fig 6. B
TrAEL-SEQ, exponential *tof1*Δ	TrAEL-SEQ, Exponential in glucose (2)	2	tof1_TrAEL_seq_rep1 (GSM7678451)<br>tof1_TrAEL_seq_rep2 (GSM7678452)	Fig 5. A-E<br>Fig 6. B
H2AP ChIP exponential *ars517*Δ*/wt*	H2AP ChIP-SEQ, Exponential in galactose with *S. pombe* (1)<br>H2AP ChIP-SEQ, Exponential in galacose (1)	2	ARS517del_exp_sp_H2AP (GSM7678377);<br>ARS517del_exp_sp_H2A (GSM7678378)/<br>wt_exp_sp_H2AP_rep2 (GSM7678365);<br>wt_exp_sp_H2A_rep2 (GSM7678366)<br><br>ARS517del_exp_H2AP (GSM7678379);<br>ARS517del_exp_H2A (GSM7678380)/<br>wt_exp_H2AP (GSM7678367);<br>wt_exp_H2A (GSM7678368)	Fig 6. C
H2AP ChIP exponential *tof1*Δ*ars517*Δ*/ tof1*Δ	H2AP ChIP-SEQ, Exponential in galactose with *S. pombe* (1)<br>H2AP ChIP-SEQ, Exponential in galacose (1)	2	ARS517del_tof1_exp_sp_H2AP (GSM7678381);<br>ARS517del_tof1_exp_sp_H2A (GSM7678382) /<br>tof1_exp_sp_H2AP_rep2 (GSM7678373);<br>tof1_exp_sp_H2A_rep2 (GSM7678374)<br><br>ARS517del_tof1_exp_H2AP (GSM7678383); ARS517del_tof1_exp_H2A (GSM7678384) /<br>tof1_exp_H2AP (GSM7678375);<br>tof1_exp_H2A (GSM7678376)	Fig 6. C
H2AP ChIP G1 arrest *wt*	H2AP ChIP-SEQ, G1 block (3)	3	wt_G1_H2AP_rep1 (GSM7678391);<br>wt_G1_H2A_rep1 (GSM7678392)	Supp. Fig. 2 I-J<br>Supp. Fig. 6 A

			wt_G1_H2AP_rep2 (GSM7678393); wt_G1_H2A_rep2 (GSM7678394) wt_G1_H2AP_rep3 (GSM7678395); wt_G1_H2A_rep3 (GSM7678396)	
H2AP ChIP G1 arrest *tof1Δ*	H2AP ChIP-SEQ, G1 block (2)	2	tof1_ G1_H2AP_rep1 (GSM7678397); tof1_ G1_H2A_rep1 (GSM7678398) tof1_ G1_H2AP_rep2 (GSM7678399); tof2_ G1_H2A_rep1 (GSM7678400)	Supp. Fig. 6 A
H2AP ChIP G1 arrest *mrc1Δ*	H2AP ChIP-SEQ, G1 block (1)	1	mrc1_ G1_H2AP (GSM7678401); mrc1_ G1_H2A (GSM7678402)	Supp. Fig. 6 A
H2AP ChIP M arrest *tof1Δ*	H2AP ChIP-SEQ, G2/M block (2)	2	tof1_M_H2AP_rep1 (GSM7678403); tof1_M_H2A_rep1 (GSM7678404) tof1_M_H2AP_rep2 (GSM7678405); tof1_M_H2A_rep2 (GSM7678406)	Supp. Fig. 6 A
H2AP ChIP M arrest *mrc1Δ*	H2AP ChIP-SEQ, G2/M block (1)	2	mrc1_M_H2AP_rep1 (GSM7678407); mrc1_M_H2A_rep1 (GSM7678408) mrc1_M_H2AP_rep2 (GSM7678409); mrc1_M_H2A_rep2 (GSM7678410)	Supp. Fig. 6 A
Copy number M arrest *tof1Δ/wt*	Copy number from G2/M block (2)	2	tof1_M_copy_number_rep1 (GSM7678413)/ wt_M_copy_number_rep1 (GSM7678411) tof1_M_copy_number_rep2 (GSM7678414)/ wt_M_copy_number_rep2 (GSM7678412)	Supp. Fig. 6 A
Copy number M arrest *mrc1Δ/wt*	Copy number from G2/M block (2)	2	mrc1_M_copy_number_rep1 (GSM7678415)/ wt_M_copy_number_rep1 (GSM7678411) mrc1_M_copy_number_rep2 (GSM7678416)/ wt_M_copy_number_rep2 (GSM7678412)	Supp. Fig. 6 A
RFA1 ChIP exponential *wt*	RFA1 ChIP-SEQ, Exponential in glucose with *S. pombe* (1) RFA1 ChIP-SEQ, Exponential in galactose with *S. pombe* (2) RFA1 ChIP-SEQ, Exponential in galacose (1)	4	wt_expglu_sp_RFA1 (GSM7678417); wt_expglu_sp_input (GSM7678418) wt_exp_sp_RFA1_rep1 (GSM7678419); wt_exp_sp_input_rep1 (GSM7678420) wt_exp_sp_RFA1_rep2 (GSM7678421); wt_exp_sp_input_rep2 (GSM7678422) wt_exp_RFA1 (GSM7678423); wt_exp_input (GSM7678424)	Supp. Fig. 6 A
RFA1 ChIP exponential *tof1Δ*	RFA1 ChIP-SEQ, Exponential in glucose with *S. pombe* (1) RFA1 ChIP-SEQ, Exponential in galactose with *S. pombe* (1) RFA1 ChIP-SEQ, Exponential in galacose (1)	3	tof1_expglu_sp_RFA1 (GSM7678425); tof1_expglu_sp_input (GSM7678426) tof1_exp_sp_RFA1 (GSM7678427); tof1_exp_sp_input (GSM7678428) tof1_exp_RFA1 (GSM7678429); tof1_exp_input (GSM7678430)	Supp. Fig. 6 A
RFA1 ChIP exponential *mrc1Δ*	RFA1 ChIP-SEQ, Exponential in glucose with *S. pombe* (1) RFA1 ChIP-SEQ, Exponential in galactose with *S. pombe* (1) RFA1 ChIP-SEQ, Exponential in galacose (1)	3	mrc1_expglu_sp_RFA1 (GSM7678431); mrc1_expglu_sp_input (GSM7678432) mrc1_exp_sp_RFA1 (GSM7678433); mrc1_exp_sp_input (GSM7678434) mrc1_exp_RFA1 (GSM7678435); mrc1_exp_input (GSM7678436)	Supp. Fig. 6 A
H2AP ChIP exponential *Tof1 627*	H2AP ChIP-SEQ, Exponential in galactose with *S. pombe* (1) H2AP ChIP-SEQ, Exponential in galacose (1)	2	tof1627_exp_sp_H2AP (GSM7678445); tof1627_exp_sp_H2A (GSM7678446); tof1627_exp_H2AP (GSM7678447); tof1627_exp_H2A (GSM7678448);	Supp. Fig. 6 C

Dataset S1 (separate file). Individual quantifications of plasmid catenation data

Southern blot quantification of plasmid catenation experiments used in figures 4 and 5. Each genetic background has a separate tab in the spreadsheet. The data used to generate boxplots is included in the Summary for Boxplots tab. Tabs prefixed with pp have been previously published in Schalbetter et al., (1) and are included here for comparison.

SI References

1. S. A. Schalbetter, S. Mansoubi, A. L. Chambers, J. A. Downs, J. Baxter, Fork rotation and DNA precatenation are restricted during DNA replication to prevent chromosomal instability. *Proc Natl Acad Sci U S A* **112**, E4565-4570 (2015).
2. N. E. Minchell, A. Keszthelyi, J. Baxter, Cohesin Causes Replicative DNA Damage by Trapping DNA Topological Stress. *Mol Cell* **78**, 739-751 e738 (2020).
3. A. Keszthelyi, Y. Daigaku, K. Ptasinska, I. Miyabe, A. M. Carr, Mapping ribonucleotides in genomic DNA and exploring replication dynamics by polymerase usage sequencing (Pu-seq). *Nat Protoc* **10**, 1786-1801 (2015).
4. N. Kara, F. Krueger, P. Rugg-Gunn, J. Houseley, Genome-wide analysis of DNA replication and DNA double-strand breaks using TrAEL-seq. *PLoS Biol* **19**, e3000886 (2021).
5. J. Baxter *et al.*, Positive supercoiling of mitotic DNA drives decatenation by topoisomerase II in eukaryotes. *Science* **331**, 1328-1332 (2011).
6. A. P. Gasch *et al.*, Genomic expression programs in the response of yeast cells to environmental changes. *Mol Biol Cell* **11**, 4241-4257 (2000).
7. S. Tanaka, J. F. Diffley, Interdependent nuclear accumulation of budding yeast Cdt1 and Mcm2-7 during G1 phase. *Nat Cell Biol* **4**, 198-207 (2002).
8. R. Westhorpe, A. Keszthelyi, N. E. Minchell, D. Jones, J. Baxter, Separable functions of Tof1/Timeless in intra-S-checkpoint signalling, replisome stability and DNA topological stress. *Nucleic Acids Res* **48**, 12169-12187 (2020).
9. J. Baxter, J. F. Diffley, Topoisomerase II inactivation prevents the completion of DNA replication in budding yeast. *Mol Cell* **30**, 790-802 (2008).
